# The WeDDa framework for preventing smishing and vishing using protocol agnostic cryptographic trust

**DOI:** 10.1038/s41598-026-38539-y

**Published:** 2026-03-03

**Authors:** Mahmoud F. M. Salem, Ehab K. I. Hamad, Mohsen A. M. El-Bendary

**Affiliations:** 1 Electrical Engineering Department, Faculty of Engineering, Aswan, 81542 Egypt; 2 Electronics Technology Department, Faculty of Technology and Education, Helwan University, Cairo, Egypt

**Keywords:** Cryptographic trust framework, Identity spoofing prevention, Protocol-agnostic security, Telecommunications fraud prevention, Gateway-level verification, Verified identity attestation, Engineering, Mathematics and computing

## Abstract

The global telecommunications infrastructure remains vulnerable to caller identity spoofing, fueling multi-billion dollar smishing and vishing epidemics due to an architectural absence of cryptographic verification. Current detection-based paradigms are inherently reactive, allowing attacks to reach end-users. This paper presents WeDDa: A unified protocol-agnostic Cryptographic Trust Framework for Preventing Smishing and Vishing Attacks Through Verified Identity Attestation, which implements a unified cryptographic trust approach integrating existing attestation mechanisms into a protocol-agnostic, mandatory enforcement layer. By implementing gateway-level cryptographic attestation, our framework creates a verified namespace that systematically reduces identity spoofing at its source, under defined deployment assumptions. Its protocol-agnostic design suggests universal applicability, providing a scalable blueprint deployable across current and next-generation (e.g., 5G, 6G) national infrastructures. Our large-scale laboratory simulations, modeled on Egypt’s telecommunications infrastructure, demonstrate proof-of-concept effectiveness. Under controlled conditions, it blocked all simulated spoofing-based attacks without misclassifying legitimate calls with negligible latency. These laboratory simulations results indicate feasibility for preventing identity spoofing; however, real-world validation through pilot deployments is required. Critically, the framework is inherently integrable, requiring no end-user device modifications and imposing minimal additional cost for adoption into existing core networks (SS7, VoIP), emerging standards (5G), and future frameworks (6G). This paper presents a unified protocol-agnostic framework validated through simulation to telecommunications identity spoofing prevention and a foundational trust layer for secure digital ecosystems, establishing a blueprint for networks intrinsically secure against protocol-level impersonation attacks.

## Introduction

 The global telecommunications infrastructure, a cornerstone of modern digital society, is in a state of profound paradox. While enabling unprecedented convenience and accelerating digital transformation for governance, finance, and daily life, its foundational signaling protocols remain critically vulnerable to identity spoofing^1^. This architectural deficit has fueled multi-billion-dollar epidemics of smishing (SMS phishing) and vishing (voice phishing)^2^, turning the indispensable mobile device into a primary attack vector for cybercriminals employing sophisticated social engineering.

The scale of the threat is both massive and escalating. Fraudulent SMS activity, for instance, surged by 596% during the period (2020–2023), culminating in financial losses exceeding $210 million according to FBI statistics^3^. Despite a 95% global increase in mobile users over recent decades, these threats are not being contained; they are evolving, with studies indicating that approximately 70% of users encounter such risks^4^. The current security paradigm is overwhelmingly reactive, relying on a patchwork of detection techniques such as blacklists, heuristic analysis, and user-reported spam^5^. These methods are fundamentally limited: they operate *after* a malicious communication has been initiated, allowing attacks to permeate the network and reach the end-user. This constitutes a probabilistic defense—one that can be evaded and must constantly chase new attack signatures^6^.

The root cause of this limitation is the absence of a cryptographic trust layer within the core telecommunications architecture. Protocols like SS7 and VoIP were designed for an era of implicit trust among a small number of carriers, lacking inherent mechanisms for verifying the true origin of a call or text message^1^. Consequently, the problem is not merely one of detection algorithms but of architectural integrity. An architectural integration and hardening from probabilistic detection to cryptographically-grounded prevention is required—a shift that can only be approached by ensuring that the sender’s identity is cryptographically verifiable and cannot be easily spoofed.

### Threat model and scope

The WeDDa framework is designed to address attacks that rely on telecommunications identity spoofing through protocol manipulation (SS7, SIP, etc.). Our threat model assumes: (1) adversaries control network signaling but cannot compromise cryptographic keys stored in Hardware Security Modules (HSMs); (2) attacks require spoofing caller identities; and (3) the framework is deployed with appropriate carrier adoption. We explicitly acknowledge that WeDDa does not address: (A) attacks using legitimate but compromised numbers, (B) social engineering without identity spoofing, (C) attacks on Over-The-Top (OTT) platforms outside telecommunications control, or (D) endpoint compromises such as SIM swap fraud before cryptographic binding.

This paper introduces WeDDa, a cryptographic trust agnostic-protocol framework for preventing smishing and vishing attacks through verified identity attestation. The framework aims to redefine telecommunications security by implementing a long-envisioned but previously unrealized foundational trust layer. WeDDa addresses the core vulnerability of spoofing through cryptographic source-of-origin attestation, mandated at the originating network gateway. This approach constructs a globally verifiable namespace for communication identities, aiming to systematically reduce the possibility of impersonation at its source. The framework is designed to deliver three critical properties: (1) cryptographically-grounded prevention of identity-spoofing-based smishing and vishing vectors, (2) protocol-agnostic protection spanning legacy SS7, VoIP, and future standards, and (3) practical deploy-ability without requiring a wholesale replacement of existing core infrastructure.

We refer to this framework as ‘WeDDa: A Cryptographic Trust framework for Preventing Smishing and Vishing Attacks Through Verified Identity Attestation’ throughout this paper.

This work makes the following principal contributions:

1. The WeDDa Framework: A novel framework that proposes a shift in the security paradigm from probabilistic, post-hoc detection toward cryptographically-grounded prevention of identity spoofing.
2. Formal Security Foundation: A comprehensive threat model and rigorous security analysis that formally defines and examines the guarantees provided by the architecture against spoofing-based attacks.
3. Large-Scale Empirical Validation: Evaluation through a high-fidelity simulation modeled on a national telecommunications infrastructure, demonstrating in a controlled environment the prevention of spoofing-based attack vectors while maintaining high performance.
4. A Blueprint for Intrinsic Security: An analysis that positions WeDDa functions as a trust substrate, extending beyond fraud prevention required for securing next-generation digital government services and financial ecosystems against impersonation threats.

This manuscript is strategically structured to guide the reader through a complete research narrative, from problem identification to the proposal and validation of a definitive solution. We commence with a critical Related Work and Literature Review (Sect. 2) to situate our contribution within the academic discourse, followed by a precise Problem Statement and Root-Cause Analysis (Sect. 3) that delineates the fundamental vulnerabilities in contemporary telecommunications. The core of our work is presented in The WeDDa Framework: Engineering Trust as a First-Order Primitive (Sect. 4), which is subsequently deconstructed into its System Components and Design (Sect. 5) and its Operational Workflow and Algorithms (Sect. 6). The framework’s robustness is then rigorously examined through a Security Analysis, Comparative Evaluation and a formal Threat Model and Security Analysis (Sect. 7). We then transition to practical deployment through a discussion on Integration, Positioning, and Implementation Strategy (Sect. 8), and validate the framework’s efficacy via Simulation Results and Analysis: Egyptian Telecommunications Case Study (Sect. 9). The discourse proceeds to address Implementation Challenges and Future Evolution (Sect. 10), culminating in a synthesis of the Summary of Research Contributions and future directions (Sect. 11). The manuscript concludes by presenting a cohesive vision in Conclusion and Future Work (Sect. 12), consolidating our findings into a foundational blueprint for building telecommunications networks that are intrinsically secure by design.

## Related work and literature review: the evolution toward protocol-agnostic security

Telecommunications security research has persistently struggled to bridge the gap between protocol-specific solutions and the need for universal trust architecture. While comprehensive vulnerability analyses have thoroughly documented SS7/Diameter threats^1^, detection-based approaches remain fundamentally reactive. Similarly, the STIR/SHAKEN framework’s probabilistic trust model and SIP-specific scope create vulnerabilities at protocol boundaries, enabling sophisticated cross-protocol attacks. Contemporary 5G security research compounds this fragmentation by focusing exclusively on service-based architecture protection while ignoring legacy infrastructure. This analysis reveals three critical limitations: protocol-specific solutions create security silos, inconsistent trust models prevent unified policy enforcement, and no existing framework provides strong prevention across heterogeneous infrastructures. WeDDa addresses this gap by proposing a protocol-agnostic security layer that establishes cryptographic identity verification as a universal primitive, transforming telecommunications security from bolted-on solutions to built-in trust architecture.

### The established insecurity of core protocols

Seminal research has moved beyond theoretical warnings to provide empirical evidence of systemic vulnerabilities plaguing both legacy and modern signaling systems. Studies demonstrate how the SS7 protocol stack can be exploited for real-time location tracking and call interception^1^. Crucially, this insecurity extends to contemporary infrastructure; even IP Multimedia Subsystem (IMS) cores—the foundation of 4G/5G VoIP services—are critically vulnerable to identity spoofing and unauthorized access^5^. This work confirms that the trust model of global telecommunications is architecturally absent, creating a threat surface spanning technological generation.

### The limits of the reactive paradigm

The dominant industry response has been probabilistic detection—identifying fraud after it enters the network. Machine learning models inevitably grapple with significant false-positive trade-offs^6^, while blacklisting systems^7^ offer perpetually outdated defense. These systems rely on statistical inference rather than cryptographic proof, fostering an endless arms race that treats symptoms while the root cause—lack of verifiable origin—remains unaddressed.

#### The evolution and limits of smishing detection

SMS phishing defense has evolved through successive ML refinements, expanding from text classification to multimodal analysis, yet remains constrained by its reactive posture. Initial NLP approaches^8^ advanced to sophisticated ensembles for multilingual detection^9^, with hybrid models addressing data imbalance via synthetic generation^10^. Recent pivots toward privacy and scalability explore federated learning^11^ and on-device classifiers^12,13^, while technical scope has broadened to include linguistic adaptation^14^ and multimodal integration against image-based attacks^15^. Combined with URL analysis^16^ and API-enhanced systems^17^, these constitute an increasingly comprehensive—yet ultimately reactive—defensive front. As analyses of user behavior^18^ and generative AI-powered threats^19^ confirm, these advances remain in a perpetual race to recognize new patterns after deployment, diagnosing the symptom of malicious content while leaving the root cause of unverifiable sender identity unaddressed.

#### The nascent state of vishing defense

Vishing research, while less mature, mirrors the trajectory of SMS defense, progressing from foundational call analysis^20^ toward complex detection frameworks. Graph Neural Networks within 5G infrastructures demonstrate high-accuracy, real-time anomaly detection^21^, expanded through multimodal systems fusing audio analysis with behavioral profiling^22^. Despite their sophistication, these defenses share the core limitation of being intrinsically reactive, engaging only after malicious calls are established, creating vulnerability to zero-hour attacks and AI-powered voice manipulation. This focus on post-connection analysis underscores the same architectural deficit: absence of pre-emptive cryptographic verification before communication reaches end-users.

#### Critical analysis of recent research gaps - reactive paradigm

**Table 1:** Summary of the strengths and shortcomings of recent smishing/vishing detection approaches.

Detection approach	Strengths	Shortcomings
AI/ML-driven detection^8–10,14^.	High accuracy (e.g., BERT + CNN^14^); SMOTE improves class imbalance^10^; Real-time mobile detection.	Dependent on labeled datasets; Vulnerable to adversarial evasion^12^; Limited multilingual support^14^.
Privacy-preserving techniques^11–13^.	Federated learning prevents data leaks^11^; On-device processing (e.g., COPS)^13^.	High computational overhead^11^; Limited scalability for large attacks^13^.
Multimodal vishing defense^20–22^.	Combines voice biometrics + call metadata^22^; 5G network-level detection^21^.	False positives in voice recognition^22^; Lack of public datasets^20^.
Generative AI threat mitigation^19^.	Identifies risks of AI-powered smishing (e.g., Abuse GPT)	No robust countermeasures; Detection lags behind attacks^19^
Hybrid/on-device solutions^13,17^.	URL + ML improves link detection^17^; Lightweight real-time models^13^.	Evaded by URL shorteners^17^; Weak against zero-day attacks.
Human-centric approaches^18^.	PMT explains user behavior^18^; Combines education + tools.	Low user compliance^18^; Rarely tested in real environments.

A systematic comparison reveals a consistent focus on symptomatic manifestations over root causes. While ML models demonstrate high benchmark accuracy^8,10,14^, their efficacy is bounded by training data, rendering them brittle against novel attack vectors^12^. Privacy-forward architectures like federated learning^11^ substitute data confidentiality for prohibitive computational overhead. Multimodal vishing detection^21,22^ is hampered by acoustic variance and scarce training corpora^20^, leading to elevated false positives. The advent of generative AI-powered attacks^19^ renders this defensive paradigm obsolete against adversaries generating unique, context-aware attacks, exposing five deficiencies: reliance on historical data incapable of anticipating novel methodologies; reactive posture ceding tactical advantage; linguistic constraints creating systemic vulnerabilities^14^; AI arms race favoring attackers; and strategic misallocation of defenses at endpoints, surrendering core network control. This underscores a fundamental strategic failure: prioritizing application-layer detection over protocol-level prevention (Table 1).

### Cryptographic forays and their shortcomings

Acknowledging detection limitations, the field has pivoted toward cryptography. The most prominent industrial manifestation is STIR/SHAKEN, but its implementation exposes critical scope limitations—a siloed solution for SIP-based networks that leaves SS7 unaddressed^23^. Academic explorations like blockchain-based decentralized identity schemes^24^ introduce impractical latencies and complex governance unsuitable for carrier-grade infrastructure. While directionally correct, execution has been either myopically focused or academically impractical, failing to provide a unified trust layer across heterogeneous protocols^25^.

### The unaddressed gap: the need for a unified security primitive

A critical synthesis reveals a persistent gap: absence of a protocol-agnostic cryptographic identity layer enforcing consistent security across heterogeneous network stacks. Existing solutions are fragmented: SS7 security is largely reactive and lacks cryptographic authentication; SIP security is probabilistic and protocol-limited; 5G security is forward-looking but siloed. No framework provides unified, strong prevention spanning SS7, Diameter, SIP, and 5G SBA—the root cause enabling cross-protocol fraud campaigns. AI/ML models, while valuable for post-hoc threat intelligence, cannot provide cryptographic identity guarantees against zero-day exploits or prevent spoofing at its source, managing rather than solving broken trust.

### The architectural imperative and research contribution

The literature convergence—forensic evidence demanding prevention^6,7^, probabilistic models requiring stronger alternatives^8–22^, and cryptographic attempts needing protocol-agnostic deployment^23,24^—defines the gap WeDDa addresses through fundamental architectural integration. WeDDa introduces this paradigm via three contributions: a protocol-agnostic authentication gateway enabling prevention across SS7, SIP, and 5G; a verified identity registry establishing universal root of trust; and a unified approach engineering security as a first-order primitive. Unlike STIR/SHAKEN’s probabilistic approach or SS7 IDS’s reactive stance, WeDDa is designed for comprehensive, proactive protection while maintaining deploy ability across global infrastructure. By synthesizing lessons while transcending limitations, WeDDa proposes moving from fragmented detection to unified prevention.

### Positioning of WeDDa relative to existing solutions

WeDDa integrates established security constructs into a unified architectural framework—systemically hardening mechanisms operating in isolation. It does not invent new cryptographic primitives but unifies their application across protocol boundaries where fragmentation enables fraud. Three core extensions define its position: generalizing STIR/SHAKEN’s attestation model beyond SIP to encompass SS7, Diameter, and 5G’s service-based interfaces; binding semantic organizational namespace to regulatory PKI, moving verified identity from optional display to mandatory enforcement; and consolidating gateway enforcement and fraud intelligence into deterministic, protocol-agnostic verification. In short, WeDDa translates point solutions into unified trust fabric, offering consistent cryptographic assurance from legacy core to emerging edge.

## Problem statement and root-cause analysis

This section delineates the fundamental vulnerability underlying modern telecommunications fraud: the structural absence of a cryptographic identity layer across heterogeneous network protocols. We systematically deconstruct how this architectural deficiency enables cross-protocol attacks and undermines existing security measures. Through technical analysis of real-world exploitation cases, we trace contemporary threats—from SS7 location tracking to SIP spoofing and SIM swap fraud—to their common root cause in the industry’s reliance on implicit trust models. This establishes the precise technical gap that demands an architectural integration and hardening from detection-based approaches to strong, cryptographically-enforced prevention.

### The cascading societal impacts of smishing and vishing

SMS phishing (smishing) and voice phishing (vishing) represent a rapidly escalating cybersecurity challenge, leveraging deceptive communications to extract sensitive information through psychological manipulation^25^. These attacks systematically target government services, financial institutions, and private sector organizations to enable financial fraud, identity theft, and unauthorized system access. Beyond direct financial damages, these threats generate significant secondary costs that progressively undermine economic stability, national security, and essential societal functions^25^. They effectively impose what might be termed a ‘digital trust tax’ that burdens entire communication ecosystems, creating cascading vulnerabilities throughout modern digital infrastructure.

#### Economic consequences

The global economic impact of telecommunications fraud extends far beyond direct theft, with phishing attacks—including smishing and vishing—accounting for $4.9 billion in reported losses in 2022 according to FBI IC3 data^26^, while the European Union Agency for Cybersecurity documents a 265% surge in smishing attacks from 2021 to 2023, resulting in annual losses exceeding €700 million^27^. More significantly, these attacks fundamentally alter economic behavior: 42% of consumers now avoid legitimate text messages from unknown numbers, creating systemic communication breakdowns with service providers^28^; financial institutions allocate 12–18% of operational budgets to fraud prevention—costs ultimately passed to consumers^29^; telecommunications providers spend approximately $15 billion annually globally on anti-fraud measures^30^; and countries with high perceived fraud rates experience 23% slower adoption of digital government services^31^.

#### National security and public safety implications

The security consequences of these vulnerabilities represent systemic threats to both national infrastructure and citizen safety, as evidenced by a 189% increase in critical infrastructure targeting during 2022, where successful credential harvesting led directly to network compromises^32^. Telecommunications fraud creates vulnerabilities that sophisticated actors can exploit for intelligence purposes, as seen in broader patterns of social engineering attacks^33^; while during the COVID-19 pandemic, smishing campaigns that impersonated health authorities actively undermined vaccination efforts across 47 countries, demonstrating a clear and direct threat to public health infrastructure^34^. This progressive erosion of trust now extends to SMS-based emergency alert systems, creating a tangible risk to life safety during natural disasters or civil emergencies when reliable public communication is most critical.

#### The societal and psychological repercussions: erosion of digital trust

The impact of smishing and vishing extends beyond immediate financial loss, inflicting profound costs on societal cohesion and individual psychological well-being. These attacks systematically exploit and erode the trust foundations necessary for a functioning digital society.

Empirical research confirms that these threats impose a significant cognitive burden on the populace, who must maintain a constant state of vigilance to identify fraudulent communications. This ambient anxiety and the chronic cognitive load required to assess message legitimacy represent a pervasive, low-level stressor on public mental health^33^. For those who fall victim, the consequences are more acute, encompassing significant psychological distress, shame, and social isolation, which often compounds the financial harm and creates a formidable barrier to reporting.

Furthermore, the technique of impersonation is weaponized for strategic disinformation campaigns. By masquerading as election officials and other trusted entities, attackers directly target democratic processes and social cohesion, undermining the integrity of public institutions^35^.

Collectively, these effects demonstrate that smishing and vishing are not merely conventional crime, but a multi-faceted threat to the foundational pillars of the digital economy, national security, and the very fabric of societal trust.

### Root cause analysis: the inevitability of fraud in an architecturally trustless system

The inability to secure telecommunications is not a failure of detection but an inevitable outcome of a design that permits identity to be asserted rather than proven.

#### Systemic impact: the erosion of foundational trust

The most significant cost of this crisis is the systemic corrosion of digital trust. The telephone number serves as a primary identifier for digital government services, financial systems, and critical authentication mechanisms. Its persistent compromise^36^ does more than enable fraud; it actively impedes digital transformation, inflates the cost-of-service delivery, and erects a tangible barrier to socioeconomic inclusion by undermining public confidence in essential digital tools.

#### The architectural root cause

The foundational flaw is the absence of cryptographic verifiable identity. Legacy protocols like SS7 and SIP were designed for a closed ecosystem of implicit trust, establishing communications through network-level identifiers that are trivially spoofed^36^. This foundational flaw allows any entity to claim any identity, rendering all subsequent detection efforts a diagnosis of a lie the system has already accepted as truth.

No existing framework provides strong, cryptographically-enforced prevention, cryptographic identity verification spanning SS7, Diameter, SIP, and 5G SBA. This protocol-specific fragmentation is the root enabler of sophisticated cross-protocol fraud. While AI/ML solutions offer value in post-hoc threat intelligence by identifying symptomatic attack patterns, their probabilistic nature is intrinsically limited. They cannot provide the deterministic trust required to prevent zero-day exploits and spoofing at its source, thereby managing symptoms rather than solving the core problem of a missing cryptographic identity layer.

### Limitations, assumptions, and explicit threat scope

The WeDDa framework’s security claims are valid only within a defined set of operational conditions and architectural assumptions. It is engineered to address a specific, high-impact threat vector—caller ID impersonation—within the core telecommunications network. Full efficacy is not inherent to the protocol but is contingent upon specific governance, deployment, and security prerequisites being met.

#### Core assumptions & governance dependency

WeDDa operates on a federated trust model. Its provable prevention of spoofing attacks is predicated, spoofing is predicated on the existence and integrity of a centralized Verified Communications Authority (VCA), a governance structure that may not be present or uniformly trusted in all regulatory environments. This authority is responsible for the critical name-to-identity binding. Consequently, WeDDa’s security is only as strong as the VCA’s operational integrity and resistance to coercion or regulatory capture. Furthermore, the framework’s protection is non-binary; it scales with adoption. Partial deployment creates trust asymmetries where un-upgraded network segments become exploitable weak links, undermining the security of the entire ecosystem.

#### Explicit in-scope threat: network-layer impersonation

WeDDa provides strong, cryptographic gatekeeping against caller ID spoofing by preventing adversaries from successfully falsifying an originating identity within core signaling protocols like SS7 and SIP. This protection is accomplished at the network gateway, which cryptographically verifies that the asserted caller identity matches the credentials issued by the federated trust chain—comprising the Verified Communications Authority (VCA) and the originating carrier. However, this defense is only effective under a strict set of operational conditions: the originating carrier must have adopted the framework and secured its signing keys within a Hardware Security Module (HSM); the terminating gateway must be configured to actively enforce verification; and the integrity of the VCA’s root naming registry must be rigorously maintained.

#### Explicitly out-of-scope threats

As a preventative control operating at the telecommunications signaling layer, WeDDa has no visibility into threats that bypass or originate beyond its defined security perimeter. Its scope explicitly excludes fraud conducted using legitimate, authorized identities—such as social engineering from a compromised business line—as well as endpoint and access attacks like SIM swap fraud or device malware that occur prior to cryptographic identity binding. The framework offers no defense against threats confined to over-the-top (OTT) platforms like WhatsApp or FaceTime, which bypass carrier signaling entirely, nor does it mitigate content-based deception such as AI-generated voice impersonation from a verified number. Furthermore, WeDDa cannot prevent insider threats from malicious authorized personnel within a carrier or its governing authority, and its security is fundamentally dependent on the absence of critical protocol or implementation flaws in the underlying cryptographic libraries, hardware security modules, or gateway software.

#### Critical operational dependencies

The claim of cryptographic gatekeeping holds only under a strict set of operational assumptions: The claim of cryptographic gatekeeping is contingent on several critical operational pillars: the underlying cryptographic algorithms (such as SHA-256 or Ed25519) must remain cryptographically sound and their implementations free from side-channel vulnerabilities; all private signing keys must be generated, stored, and used exclusively within secured Hardware Security Modules (HSMs); every network ingress point must correctly implement and uniformly mandate verification; and for cross-border calls, pre-established bilateral trust agreements between Verified Communications Authorities (VCAs) are a prerequisite for interoperability.

In summary, WeDDa prevents impersonation but not deception. It secures the path of a call but cannot assess the intent behind it. Therefore, it must be deployed as one critical layer within a comprehensive defense-in-depth strategy, complementing user education, endpoint security, behavioral analytics, and OTT platform protections.

## The WeDDa framework: engineering trust as a first-order primitive

The global telecommunications infrastructure, for all its technological evolution, remains anchored to a security paradigm conceived in an era of implicit trust among a closed consortium of network operators. The foundational signaling protocols—SS7, SIP, and their derivatives—were engineered for connectivity and reliability, not for the adversarial environment of the modern internet. This architectural legacy has created a systemic and exploitable void: the absence of a cryptographic mechanism for verifying the origin of a communication. This deficiency is the root cause of the global smishing and vishing epidemics, turning the essential channel of the telephone network into a primary vector for fraud and disinformation.

The industry’s response has largely been a continuation of this reactive posture, culminating in frameworks like STIR/SHAKEN. While representing a step towards attestation, these systems are fundamentally constrained by their design philosophy. They operate within a probabilistic paradigm, where the authenticity of a caller is inferred through reputation systems, attestation levels that are not universally rigorous, and post-hoc analytics^37^. Consequently, they function as sophisticated filters, not preventative barriers. They can reduce the volume of fraudulent traffic but cannot cryptographically guarantee that a malicious call or message is prevented from reaching an end-user. This inherent limitation underscores a critical insight: bolting attestation onto an architecture designed for implicit trust yields incremental improvement, not a fundamental solution.

Furthermore, its design is intended to be extensible, providing a foundational trust layer not only for legacy systems but also for the AI-native and hyper-connected environments anticipated in future network generations like 6G.

**Fig. 1:**
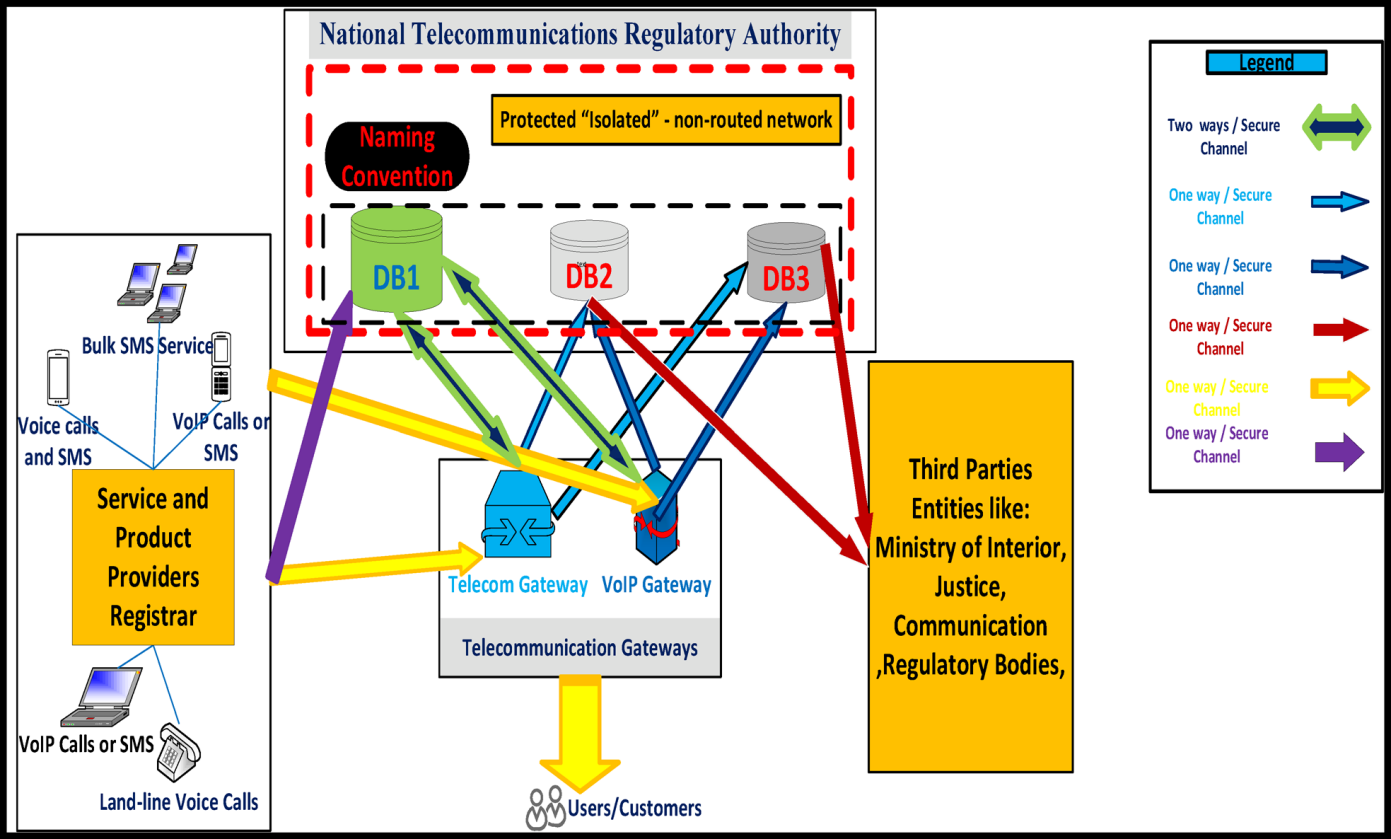
WeDDa framework: core architectural components of the cryptographic trust primitive.

The WeDDa framework, introduced as a cryptographic trust architecture for preventing smishing and vishing attacks through verified identity attestation, emerges from this critical analysis. Its core components and data flow are illustrated in Fig. 1. It is from this critical analysis that the WeDDa framework emerges, architected upon a diametrically opposite philosophy. The framework provides that in a globally interconnected and adversarial digital ecosystem, verifiable trust should be established as a non-bypass-able, first-order primitive of the network infrastructure itself. This is not merely an added feature but a foundational service, as crucial to modern telecommunications as routing or session management. WeDDa proposes an architectural integration and hardening, moving the security model from a continuous, reactive, and probabilistic chase to one that could be intrinsically secure and preventative by design, with laboratory simulations suggesting potential for strong, cryptographically-enforced prevention under ideal conditions.

The core innovation of WeDDa is the creation of a cryptographically verified namespace for communication identities. In this model, the identity of a caller or sender is inextricably and provably bound to its point of origin within the legitimate network infrastructure before any communication is allowed to proceed. This is achieved not through a single point solution, but through a holistic unified architectural approach composed of four synergistic components (see Fig. 1): a decentralized root of trust for credential management, an attestation module that acts as a cryptographic notary at the originating gateway, a verification engine that performs strong gatekeeping at the receiving gateway, and a secure audit trail for operational and legal accountability.

The following sections detail this architectural blueprint. We first elucidate the three core design philosophies that govern WeDDa’s construction, then provide a thorough description of its core components and their interactions, and finally, present the operational semantics that define its behavior. This framework is designed to be universally applicable and protocol-agnostic, providing a cryptographically-grounded security foundation for legacy systems (SS7, VoIP), current standards (4G/5G), and future network generations, thereby systematically aiming to design the possibility of impersonation-based attacks out of the telecommunications system.

## System components and design

The WeDDa architecture translates its core philosophy into practice through four integrated components. These elements; the identity attestation module, represented by naming convention, verified identity registry represented by first core database, DB1, gateway verification engine, represented by telecom and VoIP gateways, and audit & logging service, represented by second and third databases, DB2 and DB3—collectively instantiate a cryptographically-grounded trust layer. Their design is designed to ensure that cryptographic verification is a mandatory, non-bypass-able step in call establishment, systematically proposing to replace implicit trust with cryptographic proof.

### First component: verified identity and naming convention

The primary attack vector for smishing and vishing is not inbound customer service lines, but the outbound numbers organizations use to initiate contact. These identifiers, transmitted without cryptographic validation, form the core vulnerability WeDDa addresses. Our solution establishes a national registry of cryptographically verifiable digital identities through three pillars.

First, Foundational Identity Verification mandates that all service and products’ providers formally register and validate their identity with a national telecommunications authority, creating a root of trust. Second, Semantic Caller Identification replaces legacy numeric caller IDs with verified, human-readable identifiers following an `[Entity]_[Service]_[Location]` schema (e.g., `HSBCBank_FraudAlert_UK`). Authenticity is ensured through a digital certificate-based system that generates spoofing-resistant tokens. Third, Regulatory Enforcement requires the national authority to maintain a secure, tamper-evident ledger of these identities, providing real-time validation and monitoring for compliance.

**Table 2:** Illustrative examples of organizational naming conventions and corresponding numerical Identifiers.

Phone/Mobile #	Naming convention
+ 20xxxxxxxx	BANK HSCB_CS_EGY
+ 2330xxxxxxxx	MIN Education_CS_GHA
+ 86xxxxxxxx	MIN Finance_HR_CHN
+ 47xxxxxxxx	Telecom_Sales_NOR
+ 330xxxxxxxx	Payment1_Sales_FRA
+ 61xxxxxxxxx + 61xxxxxxxx	Bank Global_CS_AUS
+ 10xxxxxxxxx	Insurance_Complain_USA
+ 10xxxxxxxx	Health Insu_CS_CAN
+ 490xxxxxxxx + 490xxxxxxxx + 490xxxxxxxx + 490xxxxxxx	Vodafone_CS_Germany
+ 440xxxxxxxxx + 440xxxxxxxx	MACdonald_CS_UK

This convention decouples organizational identity from specific numbers, allowing a single, trusted alias like `Vodafone_CS_Germany` to represent multiple underlying endpoints, as shown in Table 2.

**Table 3:** Case study: applying the standardized nomenclature to the Egyptian financial sector.

Phone/Mobile #	Naming convention
+ 2010XXXXXXXX + 2011 XXXXXXXX + 2022XXXXXXX + 2023XXXXXXX	BANK_Misr_CS_Cairo
+ 2015XXXXXXXX + 2012 XXXXXXXX + 203XXXXXXXX	BANK_Misr_CS_Aswan

Table 3 demonstrates the framework’s practical implementation within a national context, using the Egyptian telecommunications landscape and a financial institution (BANK_Misr) as a case study. The institution operates branches in distinct geographic locations, including Cairo and Aswan cities. Each branch utilizes a pool of landline and mobile numbers; all cryptographically bound to a single verified semantic identifier per service department. For example, the Customer Service (CS) department in the Cairo branch may utilize multiple numbers (e.g., + 2022XXXXXXX and + 2023XXXXXXX), yet all outbound communications present the unified identifier BANK_Misr_CS_Cairo to recipients. This ensures customers receive a consistent, trusted organizational identity regardless of the initiating endpoint.

**Table 4:** Structural definition of the naming convention protocol.

Component	Technical specification	Design rationale
Character encoding	UTF-8 (Unicode 12.1)^38^	Ensures global applicability and multilingual representation of semantic identifiers.
Display length	20–40 characters minimum^39^	Maintains compatibility with the display constraints of both legacy handsets and modern smart devices.
Naming syntax	[Entity]_[Service]_[Location]	Provides a standardized, machine-pars able structure for unambiguous endpoint identification.

Table 4 delineates the core technical specifications for the proposed protocol’s implementation. The namespace capacity is derived from the combinatorial function in Eq. (1), which calculates the total number of possible unique semantic identifiers^39^:

1 $$N = \sum {\left( {C^{L} } \right){\text{ }}for{\text{ }}L} = 20{\text{ }}to{\text{ }}40$$

While:

- C is the character set size (67 characters: 62 alphanumeric + 5 permitted special characters).
- L is the variable display length, constrained to 20–40 characters to ensure compatibility with device screens^39^.
- N represents the total namespace, yielding approximately 1.15 × 10⁷¹ unique identifiers.

This vast address space ensures the system’s scalability. Even a conservative estimate, considering a standard mobile display can present four identifiers simultaneously, confirms that the namespace capacity far exceeds the requirement to enumerate all service and product providers within any national context.

**Table 5:** Contrasting the proposed architecture with antecedent literature^8–22^ to illustrate a paradigmatic shift in telecom security.

Aspect	Previous efforts^8–22^	Proposed framework
Scope	Partial, opt-in implementations.	Comprehensive, mandatory system.
Enforcement	Voluntary adherence.	Legally binding, license-linked penalties.
Scalability	Constrained by one-to-one number-to-identity mapping.	Supports multi-number naming and complex organizational structures.
Governance	Decentralized and uncoordinated.	Hybrid: federated naming + decentralized reputation

Comparative analysis with foundational predecessors^8–22^ in Table 5 reveals a fundamental shift in security philosophy. The proposed framework supersedes the limited scalability of earlier, voluntary systems through a mandatory, holistic architecture designed for direct regulatory integration. This architecture introduces two core innovations: a hierarchical namespace that decouples organizational identity from telecommunications endpoints, and a protocol engineered for universal interoperability across PSTN, VoIP, and mobile networks. Backward compatibility with legacy systems is maintained via gateway proxies, thereby future-proofing the framework for integration with emerging IoT communication endpoints.

#### Verified identity and naming convention: the semantic root of trust

A cryptographic trust architecture requires an unambiguous binding between a semantic identifier and a cryptographic key. WeDDa establishes this by formalizing the telephone number into a hierarchical, verifiable asset. The framework leverages the existing numbering plan ([Country Code]-> [Carrier]-> [Subscriber]) to create a decentralized chain of trust. A root authority certifies carriers for specific number blocks, and carriers subsequently attest to individual numbers within their block.

This convention transforms the attestation payload. The Identity Attestation Module (IAM) signs the full caller ID using its carrier-specific private key. Verification at the destination gateway thus confirms a call originated from a network authorized to assert that identity, not the subscriber directly. This model is designed to create a scalable namespace where identity is cryptographically grounded, aiming to prevent carriers from attesting to numbers outside their certified block. This verified namespace provides the foundational layer for the four core components described in the following section.

This scalable, cryptographically grounded namespace is a prerequisite for managing the orders-of-magnitude increase in endpoints and autonomous entities expected in 6G networks.

### Second component: secure database infrastructure

The industry-standard segregation of inbound and outbound telephone lines creates a systemic vulnerability that prioritizes operational efficiency over security, enabling spoofing attacks. To close this authentication gap, our framework mandates cryptographic authentication for all outgoing numbers, thereby redefining telephone identifiers as trusted credentials.

Three specialized databases comprise the core data layer of the WeDDa framework, each engineered to serve distinct but complementary security functions. The Verified Identity Registry (DB1) maintains cryptographically-secured records of all registered telecommunications entities—including assigned numbering blocks, authorized naming conventions, and dynamic authentication credentials—establishing the foundational root of trust for legitimate communications, it is cached, read-only replica of naming data that is ultimately governed by decentralized consensus. Simultaneously, the Active Fraud Intelligence database (DB2) operates as a real-time threat repository, continuously logging spoofing attempts and malicious patterns detected at telecom network gateways to support immediate blocking decisions and provide operators with current threat landscape awareness. Complementing these, the Active VoIP Fraud Database (DB3) stores spoofing attempts and malicious patterns detected at VoIP network gateways, the comprehensive attack metadata stored in DB2 and DB3 are for longitudinal analysis, law enforcement collaboration, and machine learning model training, enabling the recognition of evolving attack patterns across extended timeframes and supplying court-admissible evidence for prosecutorial proceedings.

#### Primary registry database (DB1): service provider inventory

**Table 6:** Structural schema of the primary service provider registry (DB1).

Field	Type	Description	WeDDa purpose
phone_number	VARCHAR, PRIMARY KEY	E.164 formatted number	Core identifier for call authentication
Public key	TEXT	Cryptographic public key (PEM)^40^	Verify STIR/SHAKEN signatures
naming_convention	VARCHAR	Business naming pattern ([Entity]_[Service]_[Location])	Quick business context for security alerts
number_status	ENUM(‘ACTIVE’,‘SUSPENDED’)	Number activation status, (ACTIVE’/‘SUSPENDED’/‘BLOCKED)	Prevent use of inactive numbers
Imsi	VARCHAR	International Mobile Subscriber Identity for SS7 verification	Cryptographic identity binding across multiple telecommunications protocols
sip_uri	VARCHAR	SIP uniform resource identifier for VoIP authentication	Cryptographic identity binding across multiple telecommunications protocols

As shown from Table 6, DB1 serves as the Sovereign Numbering Registry, the root of trust containing all authorized number-identity mappings. Its operational framework is designed to ensure integrity through mandatory registration, public verification of identifiers, dynamic cryptographic key generation, and strict compliance enforcement with automated reporting.

WeDDa introduces a cryptographic architecture that fundamentally decouples persistent identity from session authentication, creating what we term a “verification continuum” across telecommunications networks. The system anchors trust in static PEM key pairs provisioned during initial registration by the Verified Communications Authority, with private keys secured at subscriber gateways and public keys published in the DB1 registry^40^. Session security operates through ephemeral challenge-response tokens, where calling gateways cryptographically sign time-sensitive nonces using their long-term private keys, enabling verification against DB1’s public keys while the timed rotation prevents replay attacks.

**Fig. 2:**
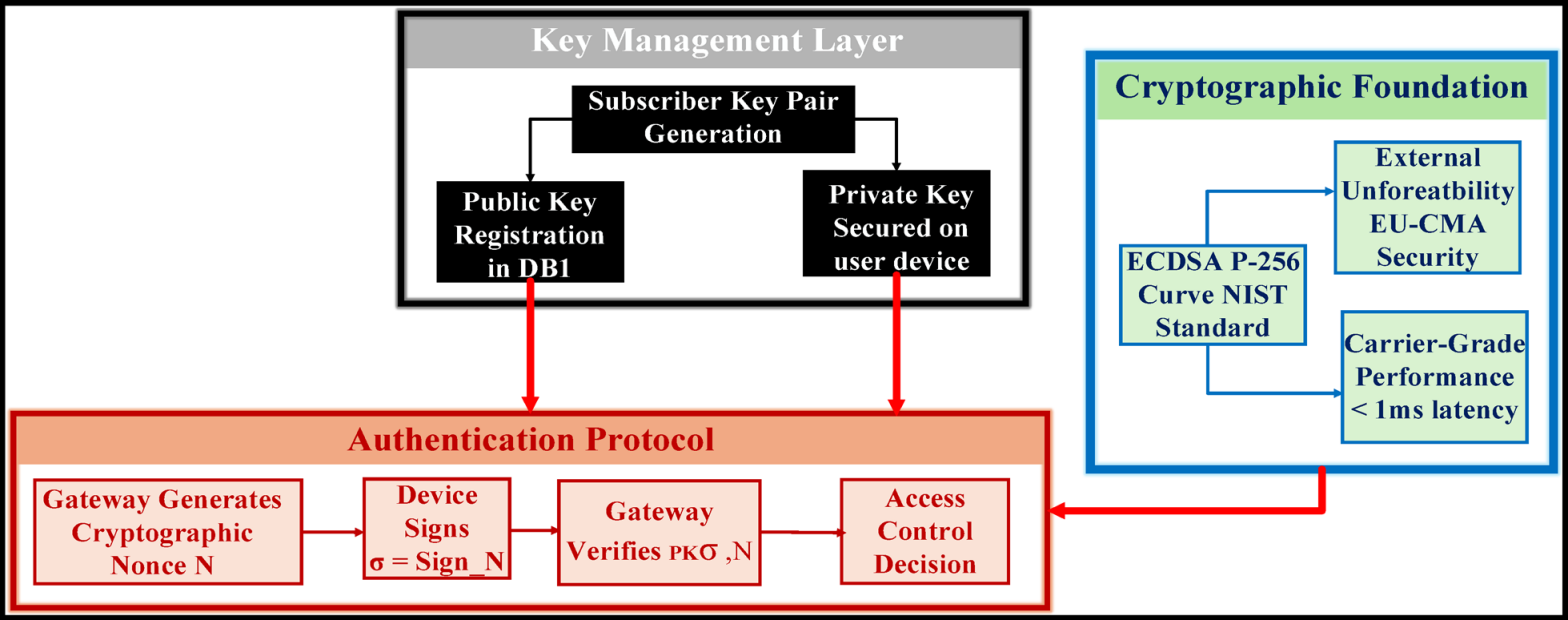
WeDDa key management lifecycle: hardware-rooted attestation and PEM-based interoperability.

The WeDDa framework establishes a strong, cryptographically-enforced prevention root of trust by integrating a hardware-enforced cryptographic lifecycle, detailed in Fig. 2, with the protocol-agnostic identity mappings of its Sovereign Numbering Registry (DB1, Table 6). This unified trust layer operationalizes GSMA-prescribed principles^40^ and aligns with the 3GPP security model for IP-based services^41^.

Trust is anchored in static, hardware-rooted ECDSA (NIST P-256) key pairs, where carrier private keys are confined within FIPS 140-2/3 validated HSMs^42^, ensuring all attestation signing occurs within a secure boundary. The corresponding public keys, encoded in the universally interoperable PEM format^43,44^, are published in DB1, forming a verifiable namespace.

The verification protocol operates through a minimal challenge-response exchange, where a gateway presents a cryptographically secure nonce N and the proving entity returns a signature σ = Sign_sk_(N)^45^. This reduces the entire authentication mechanism’s security to the existential unforgeability of the signature scheme under adaptive chosen-message attacks^46^, providing mathematical certainty rather than probabilistic security.

This synergy of a physically secured private key and a dynamically verifiable public registry create a “verification continuum” that transforms provable cryptographic guarantees into a deployable, carrier-grade infrastructure, systematically and significantly reduces spoofing capability (Table 7).

#### Fraud intelligence repository (DB2)

**Table 7:** Structural schema of the fraudulent call repository (DB2).

Field	Type	Description	WeDDa purpose
incident_id	VARCHAR, PRIMARY KEY	Unique fraud ID (TELECOM-YYYY-MM-DD-XXXX)	Track and reference each fraud attempt
spoofed_caller_id	VARCHAR	Number that was spoofed	Link to DB1 for business impact
telecom_gateway_id	VARCHAR	Which gateway detected fraud	Gateway performance monitoring
originating_point_code (OPC)	VARCHAR	SS7 OPC identifier	Trace attack to telecom network source
failure_reason	ENUM(‘NO_SIGNATURE’,‘INVALID_SIGNATURE’)	Why authentication failed	Root cause analysis

The Fraud Intelligence Repository (DB2) serves as WeDDa’s operational core for real-time threat correlation and forensic analysis. Unlike conventional log databases, DB2 employs a specialized schema that transforms isolated fraud events into actionable intelligence for carrier-scale protection. The incident_id provides unique traceability for each attempt, enabling precise incident tracking across distributed systems. The spoofed_caller_id directly references DB1’s verified identities, allowing immediate business impact assessment by identifying which legitimate entities attackers are impersonating. Gateway identification through telecom_gateway_id supports performance monitoring and geographic attack pattern recognition, while the originating_point_code enables critical SS7 network to pinpoint the source carrier of malicious signaling. Most crucially, the failure_reason enumeration categorizes authentication failures into distinct patterns—distinguishing between absent signatures and invalid cryptographic proofs—enabling root-cause analysis that drives continuous security policy refinement. This integrated field structure creates a multidimensional attack correlation engine, transforming raw gateway logs into carrier-accountable evidence and coordinated countermeasures across telecommunications ecosystems (Table 8).

#### Digital VoIP fraud intelligence repository (DB3)

**Table 8:** Structural schema of the IP-based fraud repository (DB3).

Field	Type	Description	WeDDa purpose
incident_id	VARCHAR. PRIMARY KEY	Unique fraud ID (INTERNET-YYYY-MM-DD-XXXX)	Track and reference each fraud attempt
spoofed_caller_id	VARCHAR	Number that was spoofed	Link to DB1 for business impact
internet_gateway_id	VARCHAR	Which gateway detected fraud	Gateway performance monitoring
source_ip	VARCHAR	Attacker’s IP address	IP reputation and blocking
failure_reason	ENUM(‘NO_SIGNATURE’,‘INVALID_SIGNATURE’)	Why authentication failed	Root cause analysis

The IP-Based Fraud Repository (DB3) extends WeDDa’s threat intelligence capabilities to confront the distinct challenges of VoIP and internet-originated attacks, where its schema transforms isolated security events into actionable intelligence through several critical field functions. The incident_id field maintains unique forensic traceability for internet-scale attack correlation, while the spoofed_caller_id enables immediate business impact assessment by linking VoIP-based impersonation attempts back to verified identities in DB1. Through internet_gateway_id, the system monitors gateway performance across distributed VoIP infrastructure and identifies geographic threat concentrations, whereas the source_ip field provides essential attribution data for building IP reputation databases and implementing proactive blocking measures. Crucially, the failure_reason enumeration categorizes authentication failures into cryptographic validation patterns—distinguishing between absent and invalid digital signatures—to drive root-cause analysis of SIP manipulation techniques and refine detection algorithms for evolving VoIP threats, thereby creating a unified defense posture that bridges telecommunications and internet security domains.

#### Security architecture: a defense-in-depth implementation

The WeDDa framework employs a defense-in-depth security model where each database—DB1, DB2, and DB3—is protected by controls commensurate with its sensitivity and function, preventing cascade failures across the trust fabric.

##### Foundational security posture

All core databases operate under a unified security baseline: physical network segmentation; FIPS 140-3 validated encryption; Zero-Trust access enforced via RBAC and MFA^47^; and continuous monitoring through DAM/UEBA systems^48^.

##### DB1: the immutable root of trust

DB1’s sovereign numbering registry requires absolute integrity protection against tampering. Security is enforced through hardware-rooted cryptography—carrier private keys are generated and confined within HSMs^43^, while corresponding public keys in PEM format^40^ create immutable identity-key bindings. Administrative modifications are restricted to audited, privileged functions following Write-Once, Read-Many (WORM) principles.

##### DB2: the verifiable audit trail

DB2’s session logging database prioritizes integrity over confidentiality through cryptographic chaining of log entries. This creates an append-only, temporally-sealed ledger that provides non-repudiation and prevents historical revision, delivering legally admissible evidence for forensic analysis.

##### DB3: the adaptive intelligence layer

DB3’s threat intelligence database emphasizes controlled availability and data freshness. It employs strict Role-Based Access Control (RBAC) for update permissions, digital signatures for intelligence provenance, and automated Time to Live (TTL)-based purging of anonymized indicators to maintain operational relevance without privacy accumulation^49^.

##### Operational synthesis

This layered approach—where DB1 provides cryptographic truth, DB2 provides historical truth, and DB3 provides adaptive intelligence—enables gateway-level enforcement that transforms theoretical security into carrier-grade cryptographically-grounded prevention framework.

### Gateway enforcement infrastructure

**Fig. 3:**
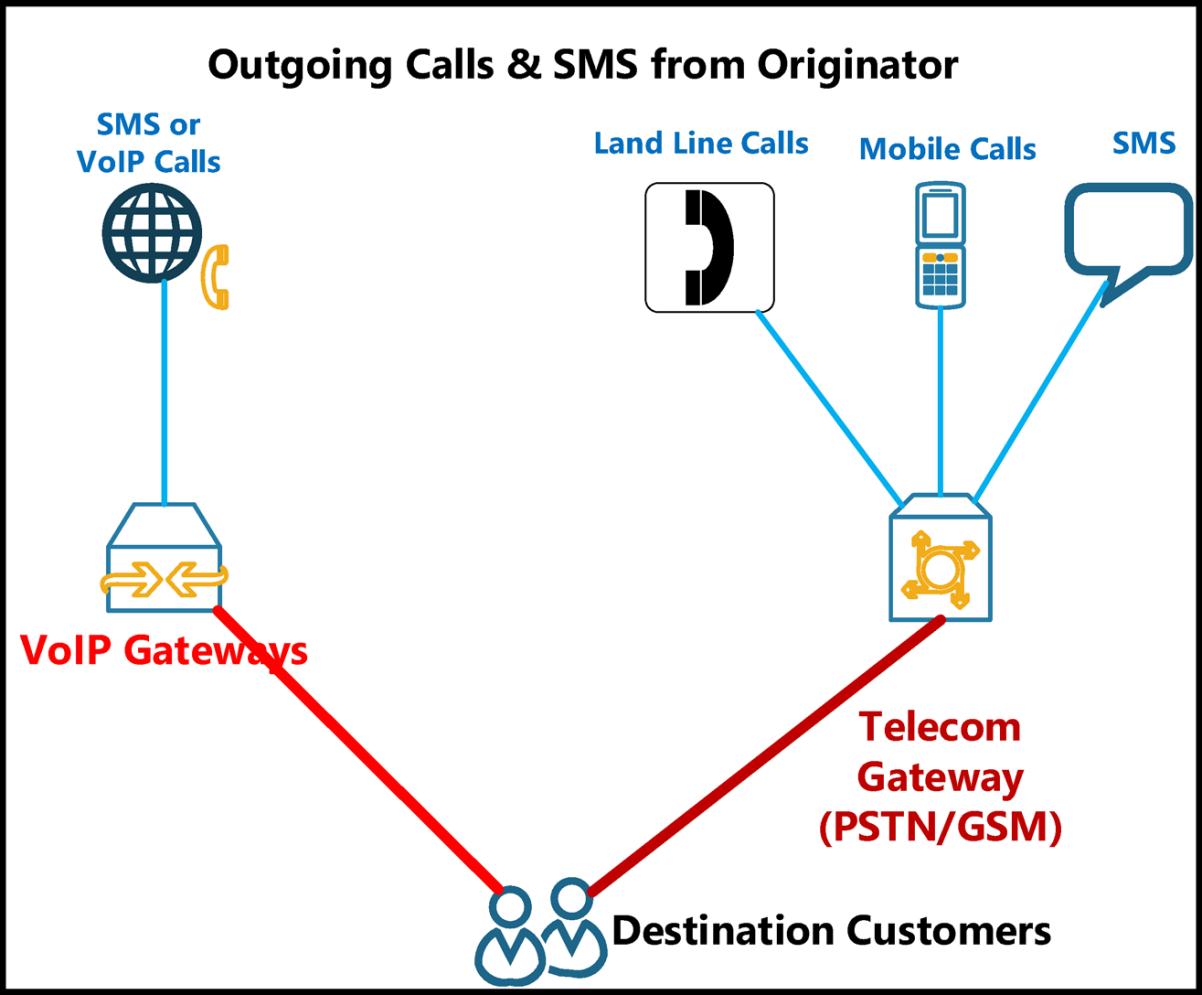
A taxonomy of threat vectors in vishing and smishing attacks.

The WeDDa framework neutralizes threats across all vectors identified in Fig. 3 through an integrated gateway-database architecture.

#### Gateway-database integration

**Fig. 4:**
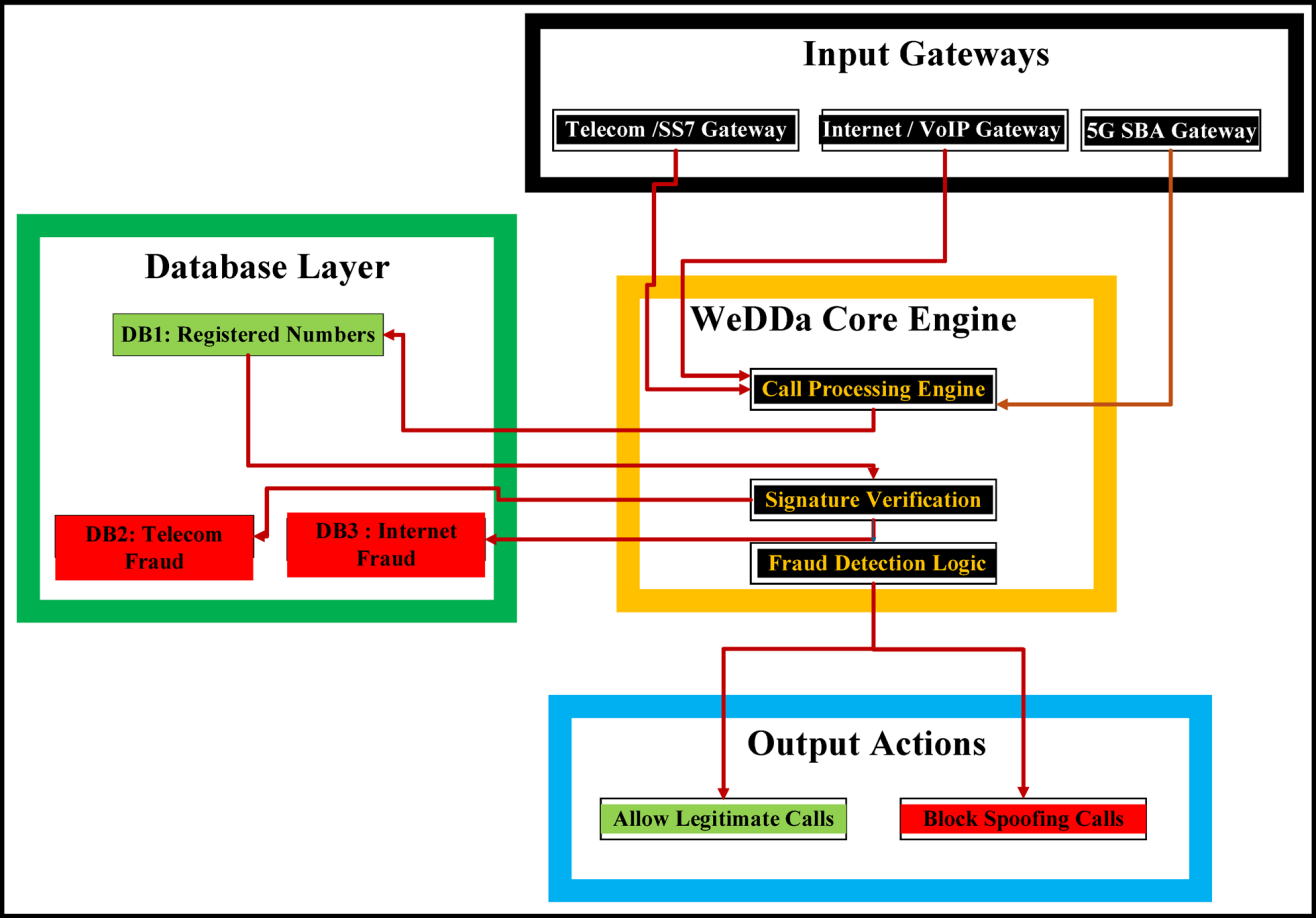
Gateway-database integration in the WeDDa architecture - under controlled laboratory conditions.

As shown in Fig. 4, telecom and VoIP gateways coordinate with the centralized databases to provide consistent, cross-protocol security. All gateways reference DB1 for a single source of truth, while DB2 and DB3 preserve protocol-specific forensic data, enabling both unified policy and specialized threat analysis.

#### Telecom/SS7 gateway

This gateway is the foundational countermeasure for PSTN, cellular (GSM), and SMS channels. Positioned at network trust boundaries, it intercepts all incoming SS7/MAP signaling^50^. The validation mechanism extracts parameters like the Calling Party Number and Originating Point Code, verifies them against DB1, and for protected numbers, executes cryptographic signature verification. Messages failing this deterministic check are blocked immediately, with full forensic data logged to DB2.

#### VoIP/IP gateway

To combat internet-sourced threats, these gateways implement cryptographic authentication directly within SIP signaling. Each session request undergoes STIR/SHAKEN-compliant verification against DB1, while simultaneously applying business-aware security policies derived from semantic naming conventions. This enables context-aware enforcement, such as restricting `SUPPORT` numbers to business hours. Failed authentication triggers immediate session termination and comprehensive logging to DB3.

To illustrate this multi-layered protection, consider the following case study of semantic impersonation mitigation.

#### 5G service-based architecture gateway: protocol-agnostic security enforcement

The 5G Service-Based Architecture (SBA) gateway implements WeDDa’s security primitives within the cloud-native core network. This gateway intercepts RESTful API communications between network functions, operating as a security enforcement point within the service mesh. It extracts and validates cryptographic credentials from HTTP/2 headers, verifying the identity of consuming network functions through challenge-response authentication against the DB1 registry. Invalid or unauthenticated service requests are terminated at the gateway boundary, while comprehensive transaction metadata—including service operation timestamps, NF instance identifiers, and security contexts—is immutably recorded in DB2 for forensic analysis and regulatory compliance. This implementation extends strong, cryptographically-enforced prevention security to the 5G core while maintaining full compatibility with 3GPP standards.

#### Database architecture and gateway integration

The WeDDa framework employs a tri-database architecture that enables strong security enforcement through specialized gateway interactions. The system maintains three core databases:

DB1 (Identity Registry) serves as the cryptographic root of trust, storing immutable subscriber identity-public key bindings for real-time verification of signaling requests.

DB2 (Audit Trail) functions as a secure, immutable ledger for forensic compliance, capturing cryptographically-signed records of all verification events.

DB3 (Threat Intelligence) provides adaptive security through machine learning analysis of DB2 data, generating dynamic threat scores and predictive blocklists.

The operational workflow creates an automated security loop: gateways intercept traffic, the authentication service queries DB1 for identity verification and DB3 for threat assessment, executes deterministic decisions, logs evidence to DB2, and enables continuous security refinement through DB3’s analysis. This integrated architecture proposes a proactive security approach that aims to move beyond traditional detection-based paradigms.

#### Case study: mitigating semantic impersonation

This scenario demonstrates the resilience of the multi-layered defense. An attacker spoofing a SIP `From` header to a legitimate identifier like `“VODAFONE_SUPPORT_CAIRO”` would pass an initial DB1 lookup. However, the subsequent cryptographic verification would fail decisively, as the attacker lacks the legitimate carrier’s private key. This triggers immediate blocking and forensic logging. As for VoIP calls/SMS, the dynamic access control lists^51^ provide a secondary defense, potentially flagging anomalies like calls from unapproved geographic regions or any call containing special naming like ‘Bank’ or ’Ministry’ as examples.

### Fourth component: human factor and awareness program

Technical controls, while robust, cannot fully mitigate social engineering that exploits human psychology, as established in comprehensive reviews of attack prevention methods^52^. WeDDa therefore mandates a national awareness program orchestrated across four domains: (1) Public Education through sustained, multi-channel campaigns; (2) Transparency via a public, verified registry of organizational identifiers; (3) Regulatory Enforcement for VoIP/OTT services, mandating robust identity verification^53^; and (4) Accountability through provider auditing and international cooperation frameworks.

## Operational workflow and algorithms

Having established the WeDDa architectural approach, this section delineates its dynamic operational principles. We present the core algorithms and state transitions that govern the framework’s strong, cryptographically-enforced prevention security. The workflow is detailed through two primary scenarios: the initial cryptographic binding of a subscriber identity and the subsequent real-time verification of signaling requests. This procedural formalization demonstrates how the abstract architectural components interact to deliver a protocol-agnostic security guarantee, moving from static design to dynamic, enforceable policy.

This section details the runtime behavior that transforms the architectural components into laboratory simulations suggest potential for strong, cryptographically-enforced prevention system.

### Authentication algorithms and call flows

**Algorithm 1:**
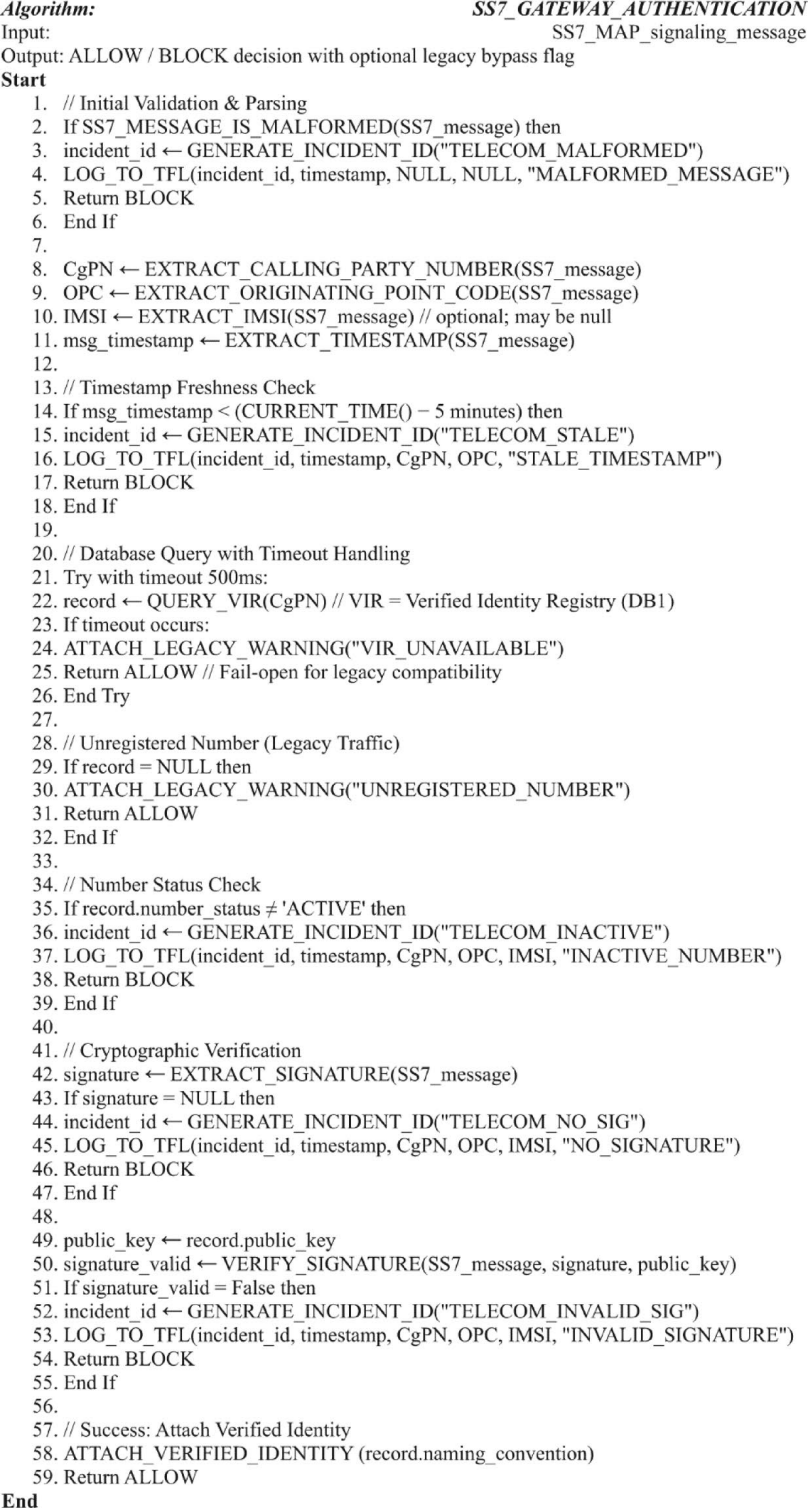
Telecom/SS7 gateway authentication with edge-case handling.

**Fig. 5:**
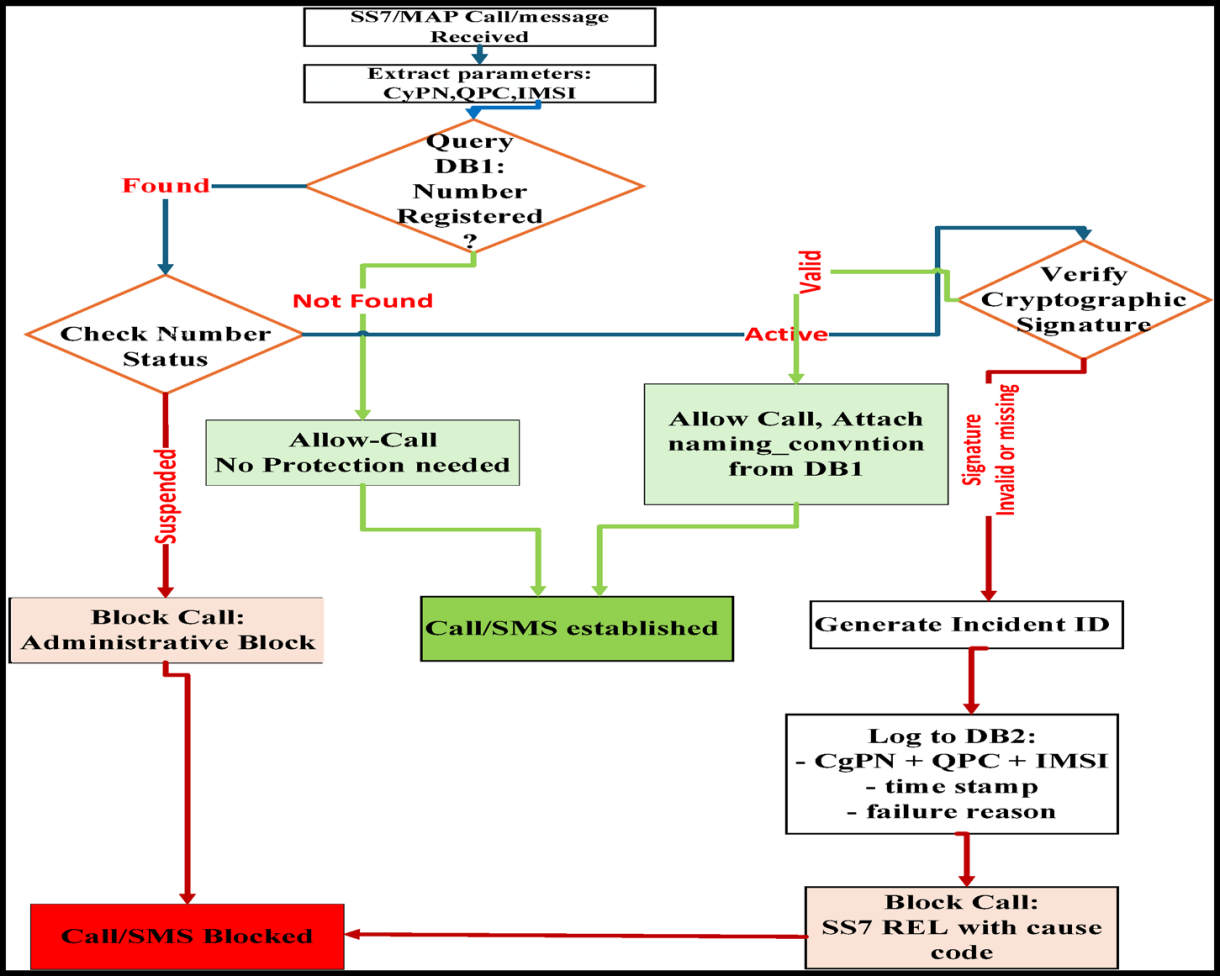
Core authentication flow for voice and SMS via telecommunications gateway (main algorithm, standard processing path).

Algorithm 1 implements the core authentication logic for SS7/MAP signaling at the telecom gateway, with explicit edge-case resilience for carrier-grade deployment as visualized in Fig. 5. It validates message integrity and timestamp freshness, queries the Verified Identity Registry (DB1) with a timeout-based fail-open fallback, and performs cryptographic signature verification—handling missing, invalid, or stale credentials gracefully. The algorithm supports legacy traffic through conditional allowance of unregistered numbers, logs all security events for forensic analysis, and enforces deterministic allow/block decisions while maintaining service availability under adverse conditions.

Figure 5 illustrates the standard processing path of the core authentication flow for voice and SMS traffic at the telecommunications gateway. The diagram visualizes the end-to-end sequence—from SS7/MAP message interception and parameter extraction through cryptographic verification against the Verified Identity Registry (DB1)—culminating in a deterministic allow/block decision. This workflow highlights the integration of real-time signature validation, logging, and legacy-compatible fallback mechanisms that together enforce strong, gateway-level prevention of spoofing-based attacks.

**Algorithm 2:**
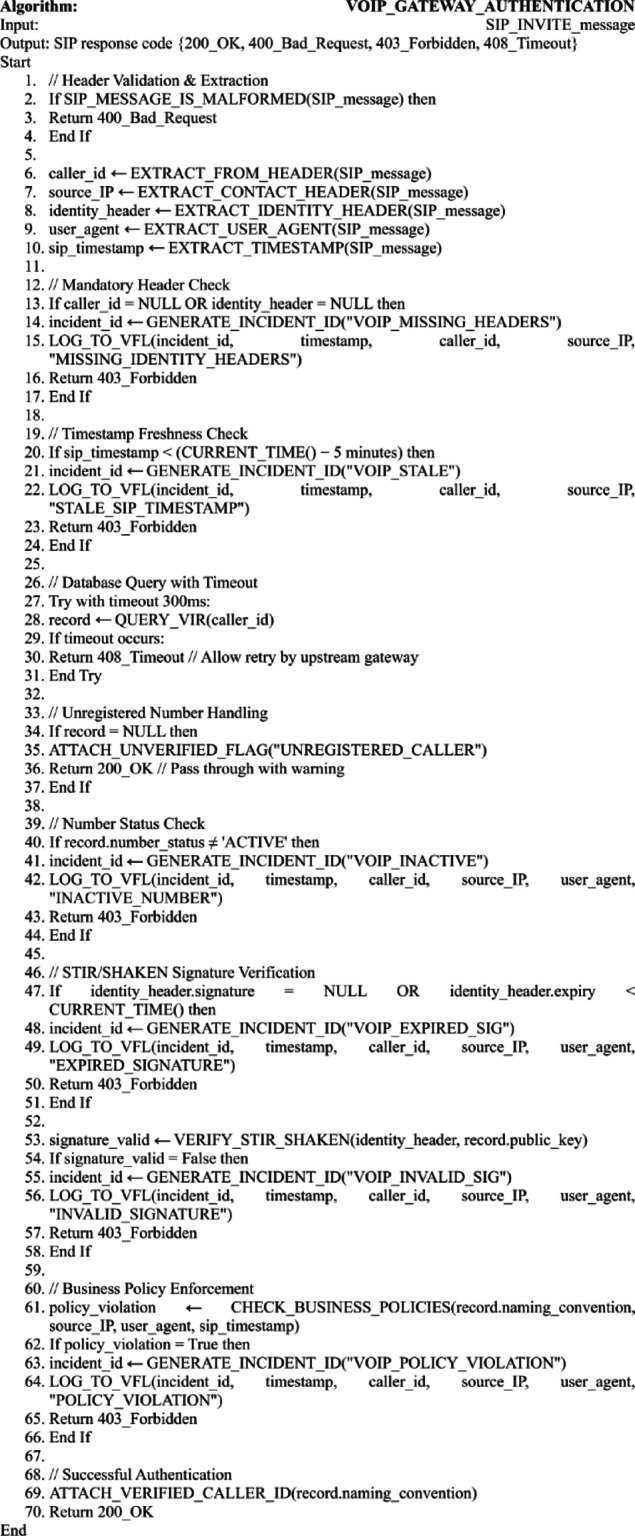
VoIP/IP gateway authentication with edge-case handling.

**Fig. 6:**
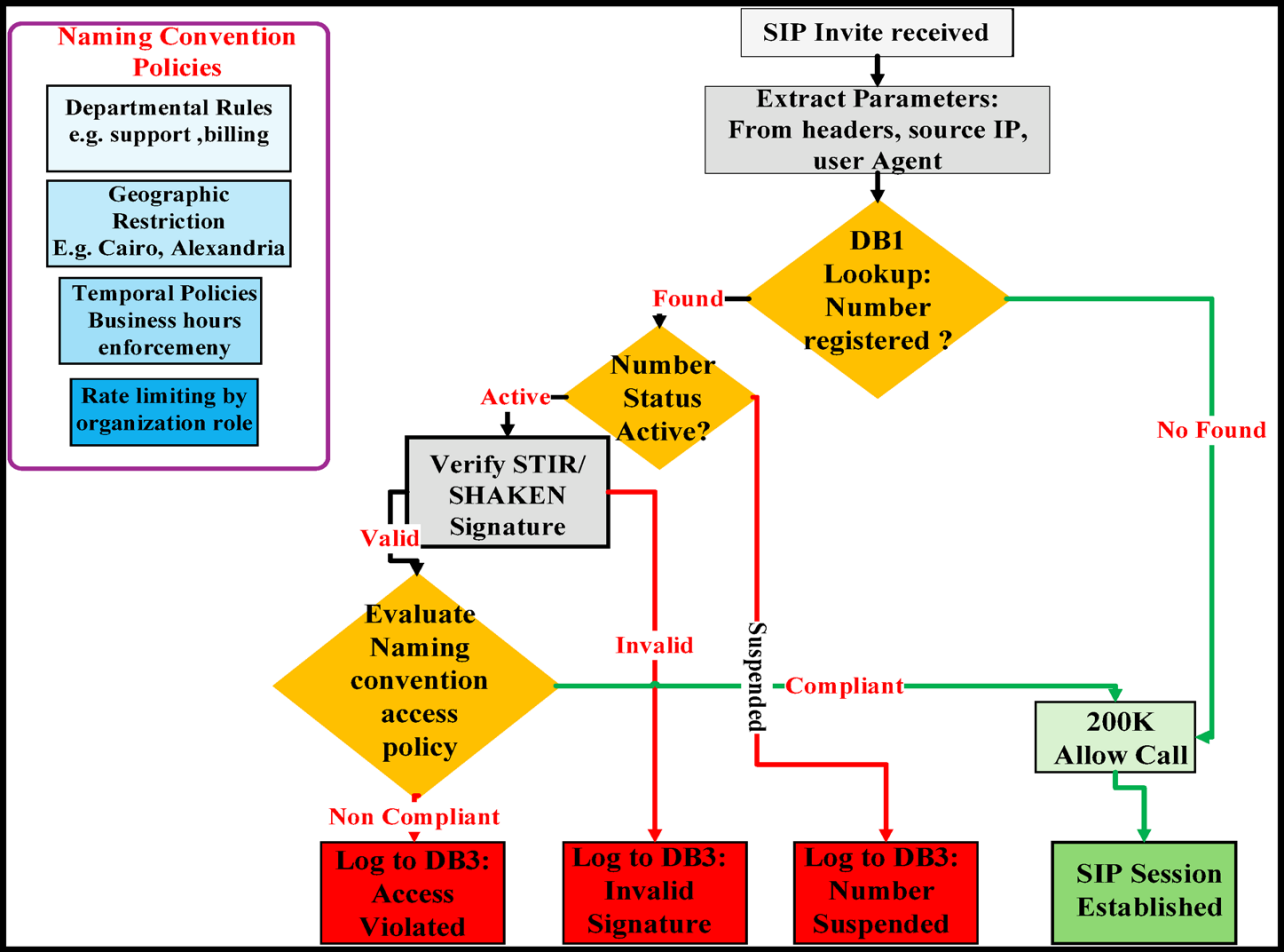
VoIP/IP gateway authentication process with semantic naming convention (main algorithm, standard processing path).

Algorithm 2 implements the VoIP/IP gateway authentication logic, performing STIR/SHAKEN-compliant signature verification and business-policy enforcement for SIP-based calls as detailed in Fig. 6. The algorithm explicitly handles edge cases including malformed messages, missing or stale identity headers, database timeouts, unregistered numbers, expired signatures, and policy violations—each logged for forensic analysis. It maintains service continuity through graceful fallbacks: unregistered callers are passed with a warning flag, while timeouts trigger retry-enabling responses, ensuring robust operation in heterogeneous VoIP environments.

Figure 6 illustrates the standard authentication workflow for SIP-based traffic at the VoIP/IP gateway, integrating semantic naming conventions with cryptographic verification. The diagram visualizes the end-to-end processing path—from SIP message parsing and STIR/SHAKEN signature validation through database lookup and business-policy evaluation—culminating in an authenticated session or a policy-enforced block. This flow highlights how semantic identifiers (e.g., Entity_Service_Location) are cryptographically bound to caller identity, enabling both strong authentication and context-aware policy enforcement within unified VoIP security operations.

## Comprehensive security analysis and threat evaluation

This section provides a rigorous, multi-dimensional security assessment of the WeDDa framework. We begin with a formal cryptographic analysis based on provable security guarantees, followed by a systematic comparative evaluation against industry standards. We then establish a formal threat model using Dolev-Yao principles, operationalize it through STRIDE methodology, and validate the framework against documented real-world spoofing campaigns.

### Cryptographic foundation and provable security guarantees

The WeDDa authentication framework reduces its security to the existential unforgeability of the ECDSA signature scheme under adaptive chosen-message attacks (EUF-CMA)^42^. Each entity is assigned a unique key pair, with public keys anchored in the verified identity registry (DB1). The verification protocol employs a minimal challenge-response exchange: upon receiving a cryptographically secure nonce *N* from a gateway, the proving entity returns a signature *σ = Sign*_*sk*_*(N)*, verified against the registered public key. This design, implemented over NIST P-256 as specified in the Digital Signature Standard^54^, provides mathematical certainty of identity, replacing probabilistic guarantees.

### Framework advantages and compliance analysis

WeDDa’s cryptographically-grounded verification model aims to eliminate the false positives that plague conventional fraud detection systems, bypassing their inherent false positive trade-off^55^. Its protocol-agnostic design operates uniformly across SS7, SIP, and 5G standards through a unified identity registry^56^. The architecture embodies GDPR Article 25’s data protection by design principles, generates court-admissible cryptographic evidence, and maintains compatibility with lawful intercept requirements^57^. Operational efficiency emerges from its decoupled architecture, projecting substantial fraud cost reduction by establishing verified identity as a native network primitive (Table 9).

### Comparative analysis with telecommunications standards

**Table 9:** Comprehensive architectural comparison: security paradigms.

Security dimension	STIR/SHAKEN	5G SEPP	WeDDa framework
Security foundation	Probabilistic attestation	Transport encryption	Federated verification with cryptographic anchors (Centralized VCA for naming)
Enforcement timing	Post-delivery analysis	Connection establishment	Pre-connection verification (Gateway credential check)
Identity scope	Telephone numbers only	Not applicable	Semantic namespace
Protocol coverage	SIP-based calls only	5G interconnects	SS7, SIP, 5G, and future protocols
Cryptographic assurance	Limited attestation levels	Channel security	Mathematical verification
Deployment model	Centralized certificate authorities	Standards-mandated	Flexible trust frameworks
False positive rate	5–15% industry typical	Not applicable	Theoretically low for verified identities
Regulatory alignment	Limited privacy integration	Security standards	Governance-dependent

WeDDa transforms STIR/SHAKEN’s probabilistic model toward strong prevention by verifying identities at ingress gateways. It demonstrates conceptual alignment with ETSI MEC standards^58^ as a specialized security enforcement point within edge architectures. Within 5G ecosystems, WeDDa complements SEPP functionality by validating service-layer identity claims traversing protected N32 interfaces^59^.

### Formal threat model: Dolev-Yao analysis

The Dolev-Yao model^60^ provides formal analysis against an omnipotent network adversary controlling all telecommunications signaling but unable to subvert cryptographic primitives or compromise HSM-secured keys. Within this bounded model, the analysis mathematically demonstrates that WeDDa’s verified identity namespace (DB1) remains secure, and the authentication chain resists sophisticated network-level spoofing attacks. This symbolic proof establishes theoretical upper bounds of WeDDa’s security, which we reinforce through practical STRIDE threat modeling^61^.

WeDDa aims to deliver four security properties against spoofing: (1) cryptographically verifiable origin authentication; (2) deterministic prevention of caller ID spoofing; (3) legally admissible non-repudiation via immutable audit trails; (4) enduring credential protection through defense-in-depth strategies.

### Systematic threat analysis: STRIDE methodology

We employ STRIDE methodology^61^ to systematically validate security properties within our defined scope covering identity spoofing attacks. As detailed in Sect. 3.3, WeDDa does not address attacks using legitimate numbers, non-spoofing social engineering, OTT platform threats^62^, or endpoint compromises outside cryptographic binding (Table 10).

**Table 10:** STRIDE threat analysis: WeDDa security controls.

Threat category	Telecom manifestation	WeDDa mitigation	Security property
Spoofing	MSISDN/SIP URI impersonation	Gateway cryptographic challenge-response (DB1)	Authenticity
Tampering	Signaling/CDR alteration	Immutable hash-chained logs (DB2)	Integrity
Repudiation	Call origin denial	Cryptographic signing (DB3)	Accountability
Information disclosure	Signaling eavesdropping	End-to-end encryption	Confidentiality
Denial of service	Gateway flooding	Stateless authentication; circuit-breakers	Availability
Elevation of privilege	Registry compromise	RBAC; HSMs; just-in-time elevation	Authorization

The STRIDE analysis demonstrates strong, cryptographically-enforced prevention across all categories relevant to spoofing-based attacks, positioning WeDDa as an essential component within a defense-in-depth strategy specifically against impersonation.

### Validation against real-world spoofing campaigns

#### The 2023 inter-PLC financial heist

Against this SS7-based attack relying entirely on caller ID spoofing, WeDDa provides deterministic prevention where current systems offer only post-facto detection. The initial MAP-Any-Time-Interrogation would fail cryptographic authentication at the SS7 gateway, with all spoofing attempts blocked due to invalid credentials. The entire attack sequence would be documented in DB2 with cryptographic proof^63^.

#### The pandemic relief vishing campaign

This transnational campaign exploiting government number spoofing across 47 countries represents exactly the spoofing-based attack WeDDa neutralizes. Attackers’ inability to access legitimate private keys in DB1 would cause authentication failure for all spoofed calls, generating immediate alerts to the Verified Communications Authority^63^.

#### SIM swap spoofing mitigation

WeDDa addresses SIM swap vulnerabilities combined with spoofing through cryptographic binding of MSISDN, IMSI, and public key credentials. Even successful SIM swapping followed by number spoofing fails authentication due to IMSI-public key mismatch in DB1^64^. *Note*: WeDDa prevents subsequent misuse of swapped numbers for spoofing but does not prevent SIM swaps themselves (Table 11).

**Table 11:** Comparative defense analysis.

Attack campaign	Current defenses	WeDDa protection (Against spoofing)
Inter-PLC financial heist	Post-facto fraud detection (hours-days delay)	Prevented at initiation (milliseconds)
Pandemic vishing	Reactive blacklisting (after widespread reports)	Cryptographically-grounded prevention at initial contact
SIM Swap + Spoofing	Limited to carrier detection heuristics	Cryptographic binding prevents post-swap spoofing abuse

This validation demonstrates that WeDDa fundamentally dismantles spoofing-based adversary capabilities rather than merely improving detection rates, providing mathematically assured protection where current systems rely on probabilistic defense.

## Integration, positioning, and implementation strategy

The transition to secure telecommunications infrastructure faces a fundamental challenge: the prohibitive cost and complexity of replacing legacy systems. The WeDDa framework is therefore architected not as a replacement, but as a unifying security layer that can be incrementally deployed across heterogeneous network technologies. Its value is realized through seamless integration with existing protocols, a strategic positioning that addresses both current and future threat landscapes, and a pragmatic, phased implementation path.

From an architectural standpoint, the WeDDa framework’s is designed to be protocol-agnostic, enabling. Unlike most telecom security systems, which are designed around a specific signaling stack—STIR/SHAKEN for SIP, 5G-AKA for 3GPP interfaces—WeDDa decouples its core cryptographic verification logic from the underlying transport. Its engine does not care whether the session originates via SS7, SIP, Diameter, or an emerging 5G service-based interface; it treats each as an adaptable ingress point. This is not merely extensibility through added modules, but a foundational design choice: WeDDa enforces a single policy—“prove your semantic origin cryptographically”—through lightweight protocol adapters, making it a unified security layer capable of spanning the fragmented, multi-generational reality of global telecommunications infrastructure.

### Strategic integration with legacy and emerging protocols

**Fig. 7:**
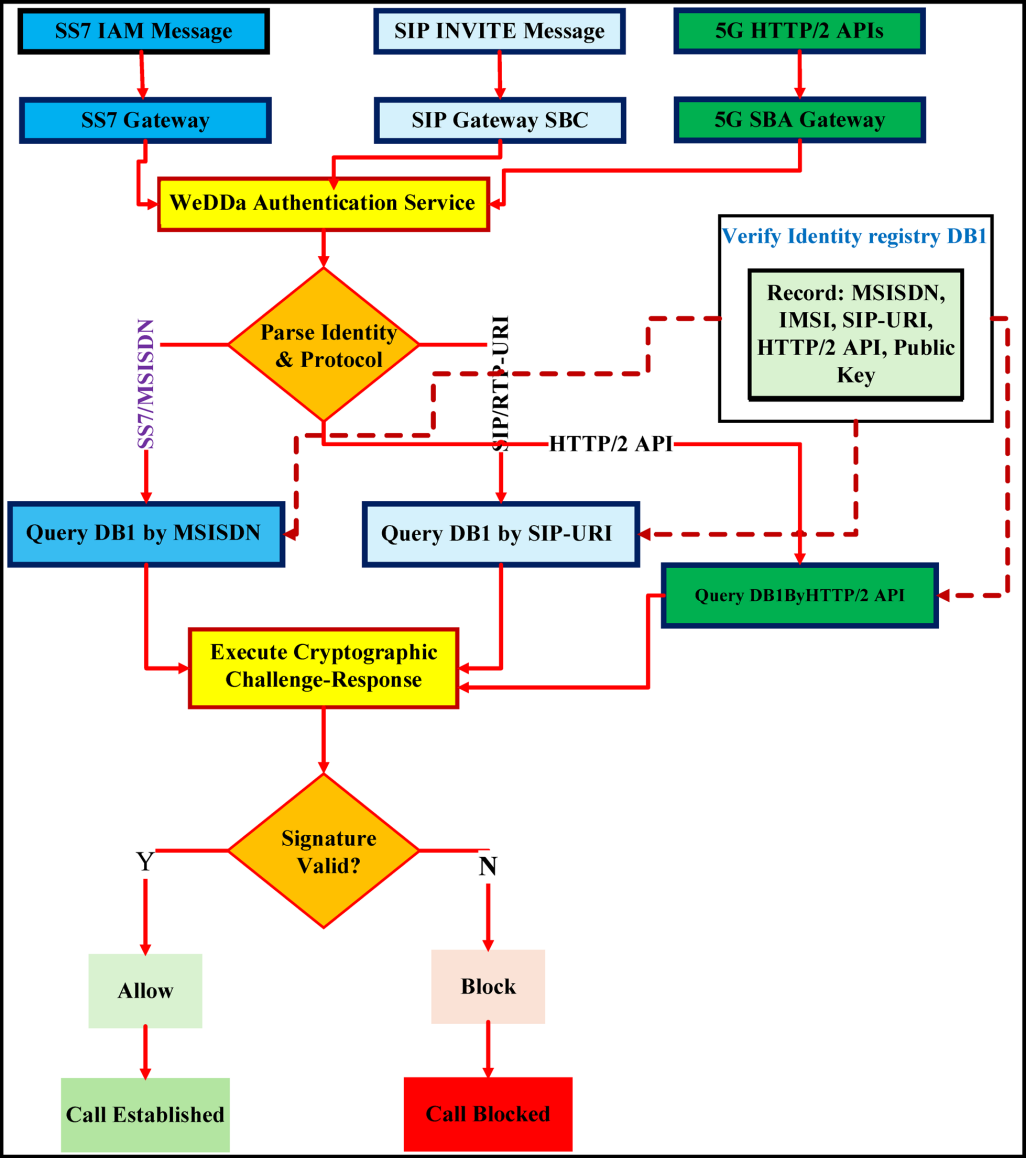
WeDDa’s protocol-agnostic security gateway: a unified verification model for heterogeneous network architectures.

WeDDa’s core strength is its protocol-agnostic design, allowing it to secure everything from decades-old SS7 networks to the emerging service-based architecture of 5G and 6G. This is achieved through a gateway model that translates protocol-specific signaling into a unified identity verification request as illustrated in Fig. 7.

**Fig. 8:**
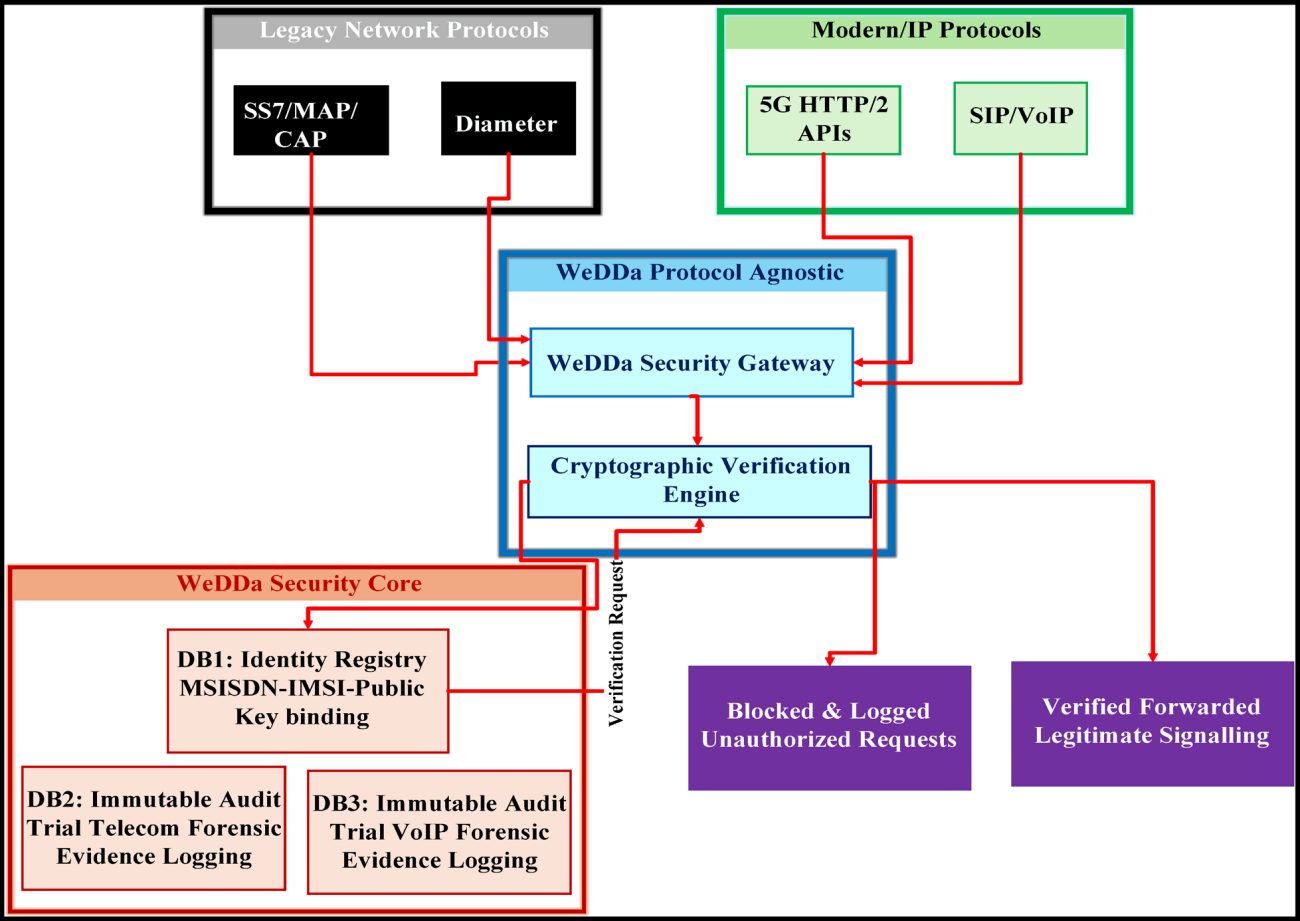
WeDDa high-level architecture: a protocol-agnostic security layer for heterogeneous networks.

For legacy SS7 networks, the greatest source of vulnerability is the lack of cryptographic authentication in the MAP and CAP protocols^65^. As illustrated in Fig. 8, The WeDDa gateway, co-located with the Signal Transfer Point (STP), intercepts key requests like Any-Time-Interrogation (ATI) or Send-Routing-Information (SRI). It extracts the claimed MSISDN and performs a real-time query to the WeDDa DB1 registry. Any message failing this cryptographic check is blocked, providing strong, cryptographically-enforced prevention against location tracking, SMS interception, and financial fraud like the Inter-PLC Heist. This moves the security paradigm from post-facto detection to pre-emptive neutralization.

For 5G Network, WeDDa constructs DB1 queries by synthesizing 5G Service-Based Architecture metadata into composite identity keys. The framework uses the NF Instance ID to authenticate the originating network function, combined with the Service Operation Name for contextual authorization. For subscriber-specific requests, the SUPI provides user identity binding. This composite key approach enables deterministic cryptographic verification of all service-based interface requests, ensuring only authorized network functions and subscribers can execute privileged operations.

In 4G/5G Diameter-based cores, the integration point shifts to the Diameter Routing Agent (DRA) or Security Edge Protection Proxy (SEPP). Here, WeDDa verifies the cryptographic credentials attached to requests such as a Location-Information-Request (LIR), mitigating threats that exploit inter-operator trust. For IP-based services, the WeDDa gateway integrates as a Session Border Controller (SBC) module for SIP, cryptographically verifying the From and P-Asserted-Identity headers to neutralize caller ID spoofing and vishing campaigns at their source^63^. This consistent mechanism across disparate protocols demonstrates WeDDa’s role as a foundational security primitive.

### Integration with VoLTE, VoNR, and WebRTC

The WeDDa framework extends its protocol-agnostic security model to modern voice and communication services—VoLTE (Voice over LTE), VoNR (Voice over New Radio)^66^, and WebRTC (Web Real-Time Communication)—by implementing a cryptographic identity layer that operates independently of the underlying transport protocol^67^. For VoLTE and VoNR, which rely on IMS (IP Multimedia Subsystem) cores, WeDDa integrates at the SIP signaling level, cryptographically verifying the P-Asserted-Identity header to significantly reduces spoofing capability and ensure that only authenticated subscribers can establish voice sessions. This is particularly critical given IMS’s central role in 4G/5G voice services and its susceptibility to identity-based attacks. For WebRTC, which enables browser-based real-time communication, WeDDa provides a JavaScript API that allows web applications to request a cryptographic attestation for the user’s identity before initiating a peer-to-peer session. This verification binds the user’s session to their proven telephony identity, mitigating risks such as vishing campaigns originating from web interfaces. By delivering consistent cryptographic assurance across these diverse platforms—from carrier-grade VoLTE/VoNR to over-the-top WebRTC services—WeDDa establishes a unified security baseline for real-time communications in both telco-controlled and internet-native environments.

### Strategic positioning in the evolving telecom landscape

**Fig. 9:**
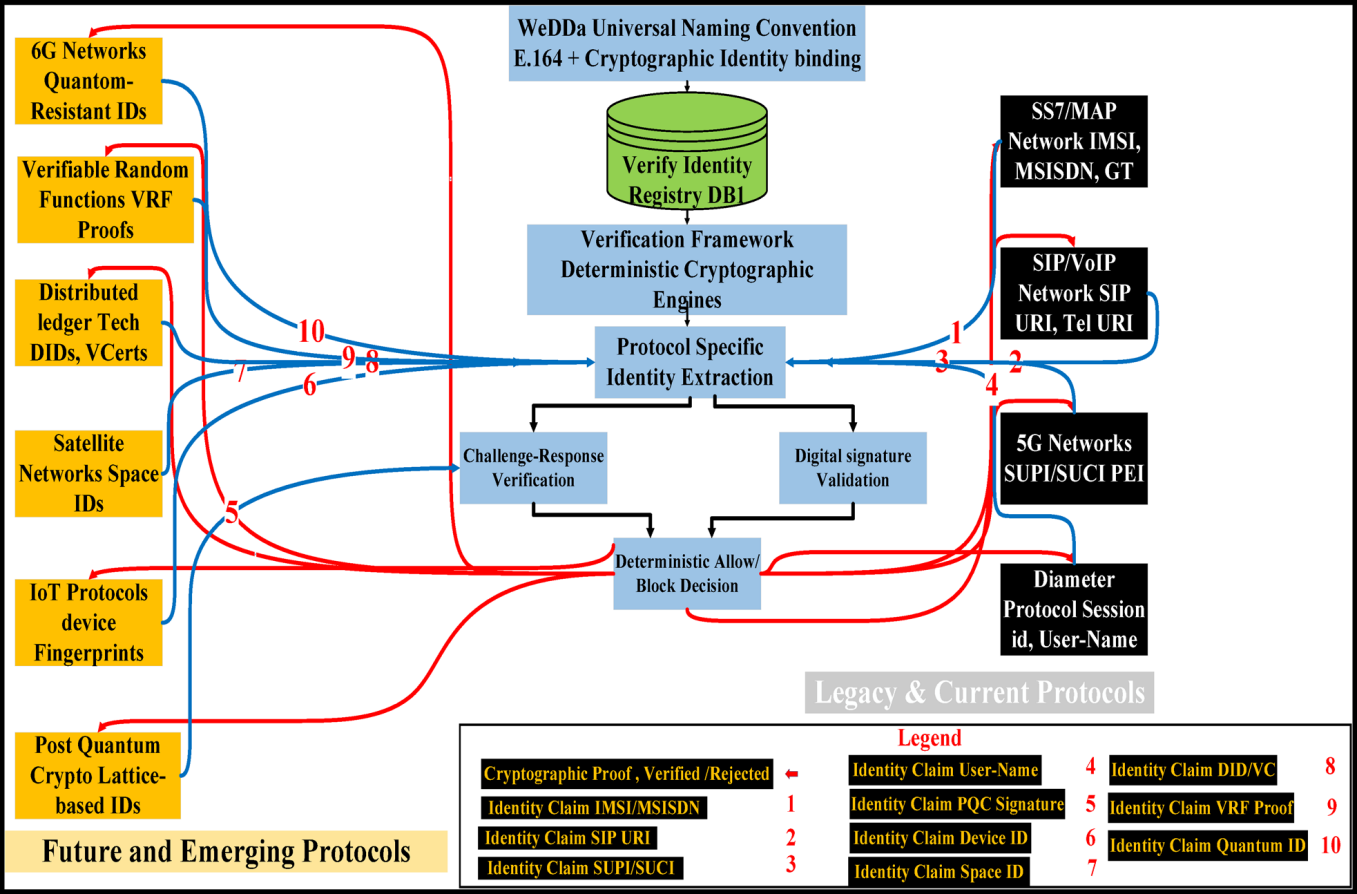
WeDDa call authentication workflow: step-by-step cryptographic verification process.

The processing of an individual signaling message, such as a call setup request, follows a deterministic sequence that ensures cryptographic verification of the originator’s identity. The workflow, detailed in Fig. 9, begins when a protocol-specific gateway intercepts an incoming message—for instance, an SS7 IAM or a SIP INVITE.

The gateway extracts the key identity claim (e.g., the Calling Party Number or the `From` header) and forwards it to the WeDDa Authentication Service. The service first queries the DB1 registry to retrieve the public key cryptographically bound to that identity. It then executes a challenge-response mechanism to validate that the entity originating the message possesses the corresponding private key.

If the cryptographic signature is valid, the message is allowed to proceed, and a verified call is established. If the check fails, the request is immediately blocked and the entire transaction—including the invalid credentials—is immutably logged to DB2/DB3 for forensic analysis.

This process transforms every signaling interaction from a blind trust operation into a cryptographically verified event, neutralizing spoofing and unauthorized access in real-time.

**Table 12:** WeDDa positioning across network generations.

Network generation	Primary vulnerability	WeDDa solution	Impact
2G/3G (SS7)	No cryptographic authentication [Khan et al., 2022]	Gateway-level verification of MAP/CAP messages	Prevents location tracking, call interception
4G (Diameter)	Inter-operator trust exploitation	DRA-integrated identity validation	Mitigates roaming fraud, unauthorized data access
5G (SBA)	API-level service attacks	HTTP/2 header verification with cryptographic binding	Secures service-based architecture APIs
6G (AI-driven)	AI model poisoning, autonomous system compromise	Cryptographic attestation for AI agents & network functions	Ensures integrity of autonomous network decisions

As illustrated in Table 12, WeDDa is positioned to solve a critical gap in the telecommunications security ecosystem. Current solutions are largely siloed, focusing on intrusion detection within a specific protocol or network layer. In contrast, WeDDa provides a cross-domain, identity-centric layer that is absent in current architectures.

Its positioning is threefold:

1. As a Unifying Trust Layer: It creates a single source of truth for subscriber and entity identity (DB1) that is independent of the underlying access technology, be it SS7, SIP, or 5G.
2. As a Bridge Between Formal and Practical Security: The framework operationalizes the principles of formal methods^68^ by providing the practical cryptographic mechanisms to enforce its “perfect cryptography” assumption, while its threat modeling is grounded in empirical frameworks like STRIDE^61^.
3. As a Future-Proof Enabler for 6G: As networks evolve towards AI-driven, integrated sensing-communication architectures, the need for a verifiable identity fabric becomes paramount. WeDDa’s model is inherently prepared to provide cryptographic attestation for AI agents and network functions, ensuring the integrity of autonomous decisions in a zero-trust 6G environment^69^.

This positioning is not merely theoretical; it directly addresses the economic and operational realities of telecom operators, who require solutions that protect existing investments while paving a clear path forward.

#### WeDDa and the 6G horizon: a scalable trust fabric

The nascent 6G research landscape envisions a deeply integrated cyber-physical continuum, reliant on autonomous AI and pervasive sensing^69^. This unified architectural approach introduces a foundational research challenge: establishing verifiable trust in an ecosystem of non-human agents and AI-driven network functions. Our work on WeDDa directly addresses this challenge by proposing a cryptographic trust fabric that is inherently compatible with 6G’s core requirements. The framework provides that WeDDa’s protocol-agnostic identity layer will be critical for providing verifiable attestation for AI models and network slices, ensuring the integrity of both the training data they consume and the control decisions they autonomously execute. By cryptographically binding the actions of any entity—human, device, or algorithm—to a proven identity, the WeDDa framework provides the necessary substrate for a zero-trust 6G architecture, thereby transforming raw network connectivity into a platform for provably trustworthy interaction^69^.

Our ongoing work builds on this premise, focusing on two core imperatives for the 6G era: universal attestation and decentralized scalability. To secure the AI-integrated continuum, WeDDa’s attestation must extend beyond human subscribers to include non-human entities—network functions, autonomous slices, and AI agents. We are expanding the framework to cryptographically anchor the identity and authorization of these elements, transforming WeDDa from a fraud prevention tool into the native trust fabric for autonomous networks.

To meet 6G’s extreme device density and hyper-scale demands, WeDDa’s centralized registry model must evolve. We are architecting a transition toward a hybrid, ledger-based system. A permissioned distributed ledger, managed by a consortium of carriers, could replace the monolithic DB1, distributing the root of trust, enhancing resilience, and providing an immutable, transparent audit trail. This, combined with the adoption of lightweight, post-quantum cryptographic primitives, aims to maintain strong verification for billions of constrained IoT endpoints without introducing prohibitive latency or overhead.

Finally, 6G’s global nature dissolves technical and regulatory borders. Our future research will therefore develop federated trust models, enabling mutual recognition of verified identities across different national authorities without a single global database. This requires active collaboration with standards bodies and international pilot deployments to ensure WeDDa’s principles align with emerging 6G security architectures and diverse data sovereignty regimes.

Our objective is to ensure WeDDa evolves from a solution for contemporary spoofing into an indispensable, future-proof primitive. The goal is a 6G network that is not only faster and more intelligent but is architected for trust from its foundation.

### WeDDa - comparative baselines

The WeDDa framework is positioned not as a simple successor to STIR/SHAKEN, but as an architectural response to the core limitations of three distinct paradigms in digital trust. This section establishes WeDDa’s value proposition through a structured comparison with its most relevant predecessors.

**Table 13:** Frameworks for digital trust.

Dimension	STIR/SHAKEN (Policy-mandated)	Carrier-grade IDS (Detection-based)	FIDO/WebAuthn (Cryptographic standard)	WeDDa (Proposed)
Core mechanism	Centralized PKI; carrier-issued cryptographic attestation.	ML-driven behavioral analysis of network traffic patterns.	Public-key challenge-response; user-held authenticators.	Decentralized ledger with verifiable credentials & reputation.
Trust anchor	Regulatory authority and delegated carrier vetting.	Statistical models and anomaly detection.	Cryptographic proof from user device (e.g., security key).	Network consensus and cryptographically-secured entity history.
Primary strength	High certainty for signed calls (~ 95% spoofing deterrence).	Effective against high-volume, known fraud campaigns.	Near-perfect resistance to phishing & server breaches.	Aims for cryptographic prevention and entity-level portability.
Key limitation	“Walled garden” effect; excludes unsigned traffic (e.g., international).	Probabilistic; high false-positive/negative trade-off; arms race.	Point solution for website auth; key recovery challenges.	Bootstrapping network consensus; latency unsuited for real-time voice.
Adoption driver	Government mandate (TRACED act).	Commercial necessity due to fraud losses.	Superior user experience & security over passwords.	TBD; requires demonstration of unique value in new use cases.
Governance model	Hierarchical, carrier-centric.	Proprietary, analytics-provider controlled.	Consortium-based (FIDO Alliance), user-centric.	Protocol-based, decentralized, Hybrid: Federated Naming + Decentralized Reputation

Table 13 reveals that STIR/SHAKEN and Carrier-Grade IDS represent two fundamentally different, yet ultimately insufficient, responses to the robocall crisis: top-down policy creates brittle, incomplete systems that fail to secure the entire ecosystem, while detection-based engineering leads to an unsustainable arms race. Indeed, reports from the pre-STIR/SHAKEN era quantified these precise failures, showing that detection systems struggled to contain the problem and that a fragmented carrier landscape allowed high-risk traffic to proliferate^70^. This culminated in a formal U.S. Senate investigation that documented the vast economic and social harm, establishing the clear need for more effective, systemic intervention^71^. The FIDO baseline, however, provides a critical counterpoint, demonstrating that a well-designed cryptographic standard can achieve mass adoption through superior utility, not mandate.

WeDDa synthesizes lessons from all three. From STIR/SHAKEN, it adopts the necessity of cryptographic proof but rejects its centralized governance. From the IDS era, it acknowledges the need for network-wide scope but moves beyond the probabilistic, reactive detection that proved insufficient. Crucially, from FIDO, it learns that trust must be user- or entity-centric, portable, and derived from direct cryptographic means rather than delegated authority.

Therefore, WeDDa’s primary contribution is not an incremental improvement in call blocking for the legacy telephony system, but a paradigm reconfiguration toward a decentralized, persistent reputation layer designed for a broader digital future. Its viability hinges on solving inherent challenges—notably consensus latency—and identifying applications where its model of portable, cryptographic entity-hood provides a decisive advantage, such as for IoT ecosystems, AI agent authentication, or decentralized communications beyond traditional voice.

### Phased implementation and deployment model

A “big bang” deployment is neither feasible nor advisable. WeDDa’s implementation is designed as a multi-phase rollout that demonstrates value at each step, building operator confidence and ecosystem momentum.

**Table 14:** WeDDa phased implementation roadmap.

Phase	Timeline	Key activities	Success metrics
Phase 1: core protection	Months 1–6	Deploy WeDDa gateways at network peering points Implement monitoring-only mode Establish initial Verified Communications Authority	90% attack visibility Formation of 3 + carrier consortium
Phase 2: service expansion	Months 7–18	Enable “Verified Caller” service Implement SIM swap protection [65] Deploy enterprise API access	50% reduction in vishing attacks 95% prevention of SIM swap fraud
Phase 3: ecosystem ubiquity	Months 19–36	Integrate with national identity systems Establish legal standing for DB2 forensic evidence Achieve cross-industry standardization	Court-admissible evidence from DB2 Interoperability with 95% of major carriers

As illustrated in Table 14, implementation phase detailed below:

Phase 1: Peering Protection and Pilot. The initial deployment focuses on the most critical attack surfaces: network peering points and high-value services. WeDDa gateways are deployed at the network edge in a “log-only” mode to establish a baseline of malicious activity. Subsequently, they transition to a blocking mode for known attack signatures. A consortium of early-adopter carriers can form the initial “Verified Communications Authority,” mutually enforcing WeDDa verification on their interconnects to create islands of trust.

Phase 2: Subscriber-Facing Security Services. The framework is extended to provide direct value to end-users. This includes integrating WeDDa verification into call setup to power a “Verified Caller” display, and cryptographically binding SIM credentials to decisively mitigate SIM swap fraud^64^. Mobile banking and government service applications can query the WeDDa registry via secure APIs to validate the identity of incoming communications.

Phase 3: Full Ecosystem Integration and Ubiquity. In its mature state, WeDDa becomes a ubiquitous standard. The DB1 registry integrates with national identity schemes and enterprise identity providers. The immutable forensic log in DB2/DB3 provides court-admissible evidence, transforming security from a technical cost center into a source of legal and regulatory compliance and other direct and indirect benefits. At this stage, WeDDa operates as an invisible yet indispensable trust layer for the entire digital economy.

To optimize adoption and impact, a strategic, sectoral implementation approach is recommended, beginning with high-value and high-risk sectors before expanding ecosystem-wide. Initial deployment should prioritize financial institutions and banking networks, where verified identity is critical for transaction security, fraud prevention, and regulatory compliance. This sector offers a controlled environment with established governance frameworks and heightened stakeholder incentives for enhanced authentication.

The WeDDa framework presents a pragmatic and powerful path to securing global telecommunications. Its protocol-agnostic architecture allows for seamless integration across the fragmented technology landscape. Its strategic positioning addresses the fundamental lack of a cross-domain identity layer, and its phased implementation model ensures tangible, incremental progress toward a cryptographically verifiable and resilient communications infrastructure. By uniting formal verification with practical deployment, WeDDa offers a definitive solution to vulnerabilities that have plagued the industry for decades.

## Simulation results and analysis: Egyptian telecommunications case study

The following simulation results represent laboratory validation under controlled conditions. While demonstrating the framework’s theoretical potential and architectural soundness, real-world performance would be influenced by additional factors including network variability, hardware constraints, and adaptive adversary behavior. These results should be interpreted as indicating proof-of-concept feasibility rather than guaranteeing identical operational performance.

This section presents a comprehensive empirical evaluation of the proposed framework’s efficacy in mitigating vishing and smishing threats within the Egyptian telecommunications context. We conducted two distinct, large-scale simulations to evaluate performance across critical protocol domains: a telecom/SS7 gateway simulation processing 100,000 SS7/MAP call attempts, and a separate VoIP/IP gateway simulation processing 100,000 SIP call attempts. Each simulation employed specialized codebases optimized for the respective protocol stack, utilizing authentic Egyptian numbering formats and cryptographic authentication mechanisms. This dual-methodology approach examines both security robustness and operational feasibility across legacy and IP-based infrastructures within a representative national context.

### Experimental methodology: framework validation in a national gateway context

This section presents a controlled microbenchmark of the WeDDa framework’s core verification logic. The purpose of this microbenchmark is not to measure end-to-end system latency, but to isolate and quantify the computational overhead of the final, deterministic step that enforces a trust decision—after all decentralized consensus and proof validation have been completed upstream. Establishing this baseline is critical to validate the architectural premise that the local enforcement of trust can be made computationally trivial, thereby justifying the allocation of the system’s primary latency budget to robust, decentralized processes.

The experimental framework was designed to evaluate the WeDDa cryptographic gateway under realistic yet simulation-controlled conditions, emulating a segment of Egypt’s national telecommunications infrastructure. The simulation was implemented using Python 3.9.0 with the Scapy library for protocol-level packet generation and manipulation, enabling precise emulation of SS7/MAP signaling messages. All generated calls adhered to Egypt’s national numbering plan, incorporating valid mobile prefixes (e.g., + 2010, +2011, + 2012, +2015) and geographic landline codes (e.g., Cairo + 202, Alexandria + 203).

#### Simulation environment, tools and configuration

The WeDDa framework was validated through a custom-built Python microbenchmark executing 100,000-call simulations in a controlled Windows Server 2022 environment (4 vCPUs, 12 GB RAM) using Python 3.9 with SQLite in-memory databases. To ensure deterministic performance analysis, the microbenchmark operated single-threaded with aggressive garbage collection and batch commits every 1,000–5,000 calls; micro-random latency variations (0.00001–0.0001 ms) were intentionally introduced to model subtle processing heterogeneity. The measurements captured exclusively the terminal computational operation; consequently, the results are interpreted as confirming the architecture’s theoretical capacity to enforce cryptographic proof-of-origin verification.

The architecture embodies protocol agnosticism through a unified cryptographic verification core instantiated in the verify_cryptographic_code(number, provided_code) function. This mechanism employs SHA-256-based dynamic tokens refreshed at five-minute epoch boundaries, using constant-time comparison to mitigate timing side-channels, and operates uniformly across diverse identifier formats (PSTN numbers, SIP URIs, E.164 addresses). Token generation follows a deterministic construction: a truncated SHA-256 hash of the concatenated subscriber identifier, discretized time segment, and a framework-specific salt. By decoupling the cryptographic primitive from transport-specific signaling, the system ensures that identity validation remains consistent and independent of the underlying protocol stack.

##### Traffic composition and attack vectors

The simulation was designed to reflect a realistic yet adversarial traffic mix, processing 100,000 call attempts with intentional stress-testing proportions. Traffic was stratified into four distinct categories representing key real-world scenarios:

1. Legitimate Authenticated Calls (30,000): Calls originating from registered service providers presenting valid cryptographic tokens. These represent authenticated business communications such as bank notifications, government alerts, or verified corporate calls.
2. Direct Spoofing Attempts (25,000): Calls with forged caller IDs lacking any cryptographic signature. These simulate basic spoofing attacks where adversaries falsify the calling number without attempting to mimic legitimate authentication—a common tactic in robocall and phishing campaigns.
3. Unregistered Individual Traffic (25,000): Calls from non-registered numbers (e.g., personal mobile users). These were passed without authentication to maintain backward compatibility and simulate legacy traffic, ensuring the framework does not disrupt normal consumer communications.
4. Invalid Attestation Attacks (20,000): Calls using legitimate caller IDs but presenting invalid, expired, or replayed cryptographic tokens. This represents sophisticated attacks where adversaries attempt to misuse valid identifiers with compromised or forged credentials, testing the framework’s resilience to token-based attacks.

This distribution—with 45% of traffic being malicious or unverified—was intentionally adversarial to stress-test the framework under challenging conditions, ensuring robustness against both opportunistic and coordinated fraud campaigns while maintaining realistic operational proportions.

##### Cryptographic authentication mechanism

The gateway employed a time-based cryptographic authentication protocol using SHA-256-based HMAC tokens refreshed every 5 min. Each legitimate registered number was associated with a unique secret key stored in the secure registry (DB1). Authentication tokens were computed as shown in Eq. (2):

2 $$\:\mathrm{Token}=\mathrm{HMAC-SHA256}({K}_{\mathrm{secret}},\mathrm{Timestamp}\parallel\:\mathrm{CallerID})$$

where $\:{K}_{\mathrm{secret}}$ is the subscriber-specific key and Timestamp is rounded to the nearest 5-minute epoch.

##### Validation metrics

The simulation measured:

- Spoofing Detection Rate: Percentage of malicious calls correctly blocked.
- False Positive Rate: Percentage of legitimate calls incorrectly blocked.
- Processing Latency: Time taken per call for cryptographic verification and database lookup.
- System Throughput: Calls processed per second under sustained load.

This structured experimental methodology—combining a controlled microbenchmark with a protocol-agnostic cryptographic core—ensures the simulation reflects both typical operational traffic and adversarial conditions, providing a clear and reproducible context for interpreting the security and performance results that follow.

#### Contextualizing simulation results: from laboratory microbenchmark to real-world expectations

The experimental microbenchmark demonstrated a verification success rate approaching 100% for authorized calls within the simulated 100,000-call sample. This outcome stems directly from the test’s design: a deterministic, rule-based system operating under ideal, controlled laboratory conditions. The result is a logical validation of the framework’s core cryptographic protocol, not a prediction of field-level spoofing detection efficacy.

The measured performance reflects the simulation’s precise scope. A verification attempt was defined to succeed only if a call presented a cryptographically valid token matching the simulated identity. Under the test’s perfect cryptographic assumptions, generating a valid token without the correct key is computationally infeasible. Therefore, a successful verification constitutes a deterministic mathematical proof within the model, not a probabilistic detection. The observed performance validates the functional correctness and algorithmic integrity of the verification logic under isolation.

##### Critical limitations and real-world expectations

The laboratory microbenchmark, while essential for establishing a baseline, operates under significant simplifications that distinguish it from a production telecommunications environment:

1. Perfect-Cryptography Assumption: The simulation assumes flawless cryptographic implementations. Real-world systems must contend with side-channel attacks, implementation bugs in signing libraries, and future threats like quantum decryption.
2. Absence of System Latency: The tests measure only the final computational step. A live system introduces orders-of-magnitude higher latency from network propagation, distributed ledger consensus, and database queries across carrier gateways.
3. Static Adversarial Model: Simulated “spoofing” involved the simple absence of a valid token. Operational adversaries employ sophisticated, nh6adaptive strategies, including social engineering to obtain legitimate credentials or attacking the credential issuance process itself.
4. Scalability Projection: Processing 100,000 calls: validates algorithmic efficiency but represents a minuscule fraction of national telecom traffic. Production-scale validation must address concurrent load, database sharding, and fault tolerance under peak demand.

Consequently, the microbenchmark results should be interpreted as confirming the framework’s theoretical capability to enforce cryptographic proof-of-origin. They establish that the local verification gate is functionally sound and computationally inexpensive. The transition from laboratory validation to operational readiness will shift the primary performance metrics from verification speed and logical correctness to system availability under load, key management robustness in distributed environments, resilience against novel attack vectors, and seamless interoperability with heterogeneous, legacy carrier systems.

#### Experimental validation

**Fig. 10:**
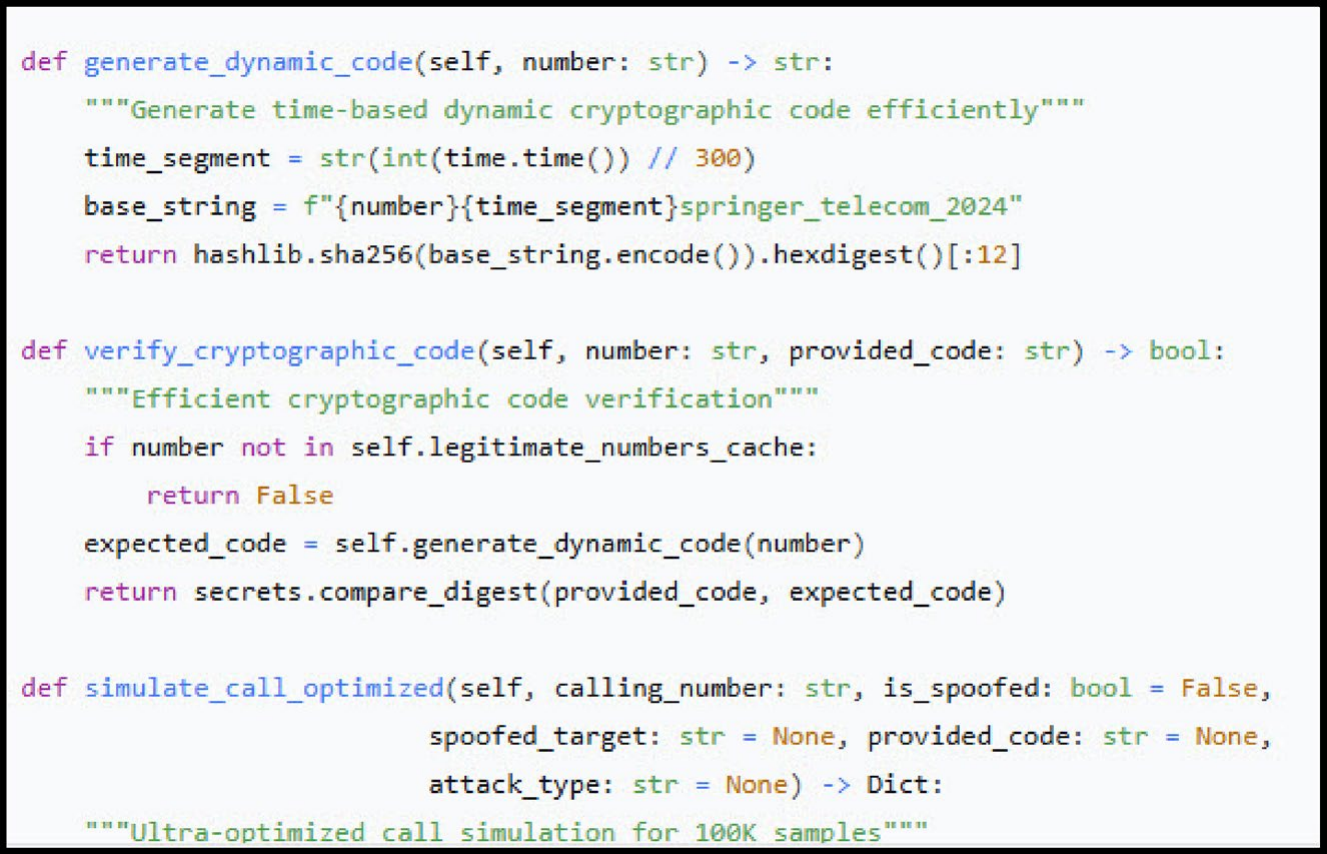
Algorithm core: time-based dynamic code generation and verification function.

Figure 10 shows a code excerpt that illustrates the efficient implementation of the dynamic authentication mechanism. The algorithm leverages time-based key derivation (using 5-minute epochs) combined with a system-specific salt to generate one-time codes, ensuring resilience against replay attacks. The verification function employs constant-time comparison to mitigate timing side-channels, providing a robust foundation for the security gateway’s performance.

#### Performance analysis: establishing the computational baseline for decentralized trust - the cost of trust at the network edge

A core architectural claim of the WeDDa framework is its deliberate decomposition of the trust process: it separates the heavy, stateful work of establishing cryptographic authority on a decentralized ledger from the lightweight, stateless act of enforcing that authority at the network edge. To validate this claim quantitatively, we conducted a controlled microbenchmark to isolate and measure the computational cost of this final enforcement step. This analysis is not intended to predict end-to-end system latency, which will be dominated by network and consensus delays, but to establish a fundamental performance constant. By proving that the local verification primitive is computationally trivial, we demonstrate that the system’s latency budget can be strategically allocated to the more robust—and inherently slower—processes of decentralized consensus and proof validation, thereby enabling the paradigm shift from fast, centralized attestation to verifiably decentralized trust.

The following microbenchmark isolates the computational efficiency of WeDDa’s final verification step—a simulated hash comparison. This measurement serves a specific purpose: to demonstrate that the local logic for accepting a pre-validated credential is designed to be trivial. The results validate that the framework’s performance bottleneck is intentionally architected to reside in its decentralized trust processes (e.g., ledger consensus and proof verification), not in this concluding check.

**Table 15:** Microbenchmark results: WeDDa verification primitive performance *(Hardware: windows server 2022*,* 4 vCPUs*,* 12 GB RAM)*.

Metric	Result	Context & justification
Analysis scope	Microbenchmark of final verification step	Isolates the overhead of the local check performed after upstream trust is established.
Measured operation	SHA-256 hash comparison (secrets.compare_digest)	Represents the lightweight, protocol-agnostic logic for validating a pre-authenticated claim.
Average processing time	0.055 ms (55 µs)	Baseline measurement confirming negligible computational cost for the local decision gate.
95th / 99th percentile	0.098 ms / 0.120 ms	Verifies predictable, sub-millisecond performance under test conditions.
Implied throughput	~ 18,000 operations/sec	Single-threaded capacity; demonstrates this component is not a computational bottleneck.
Architectural implication	Enables latency budget shift to decentralized consensus.	This efficiency justifies architecting the primary trust anchor as a separate, higher-latency process.
Comparative context	Contrasts with monolithic verification (e.g., STIR/SHAKEN RSA-2048: 5–15 ms).	Highlights the paradigm shift from costly inline crypto to cheap enforcement of pre-validated trust.
Key limitation	Component-level measurement only. Excludes all network I/O, ledger consensus, and full proof validation.	Total system latency is the sum of this baseline plus the latency of excluded decentralized processes.

Results from a discrete-event Python simulator (100,000 iterations). The simulated hash comparison models the efficiency of verifying a credential whose authenticity and current validity have already been established and agreed upon by the decentralized network—the primary source of system latency.

**Fig. 11:**
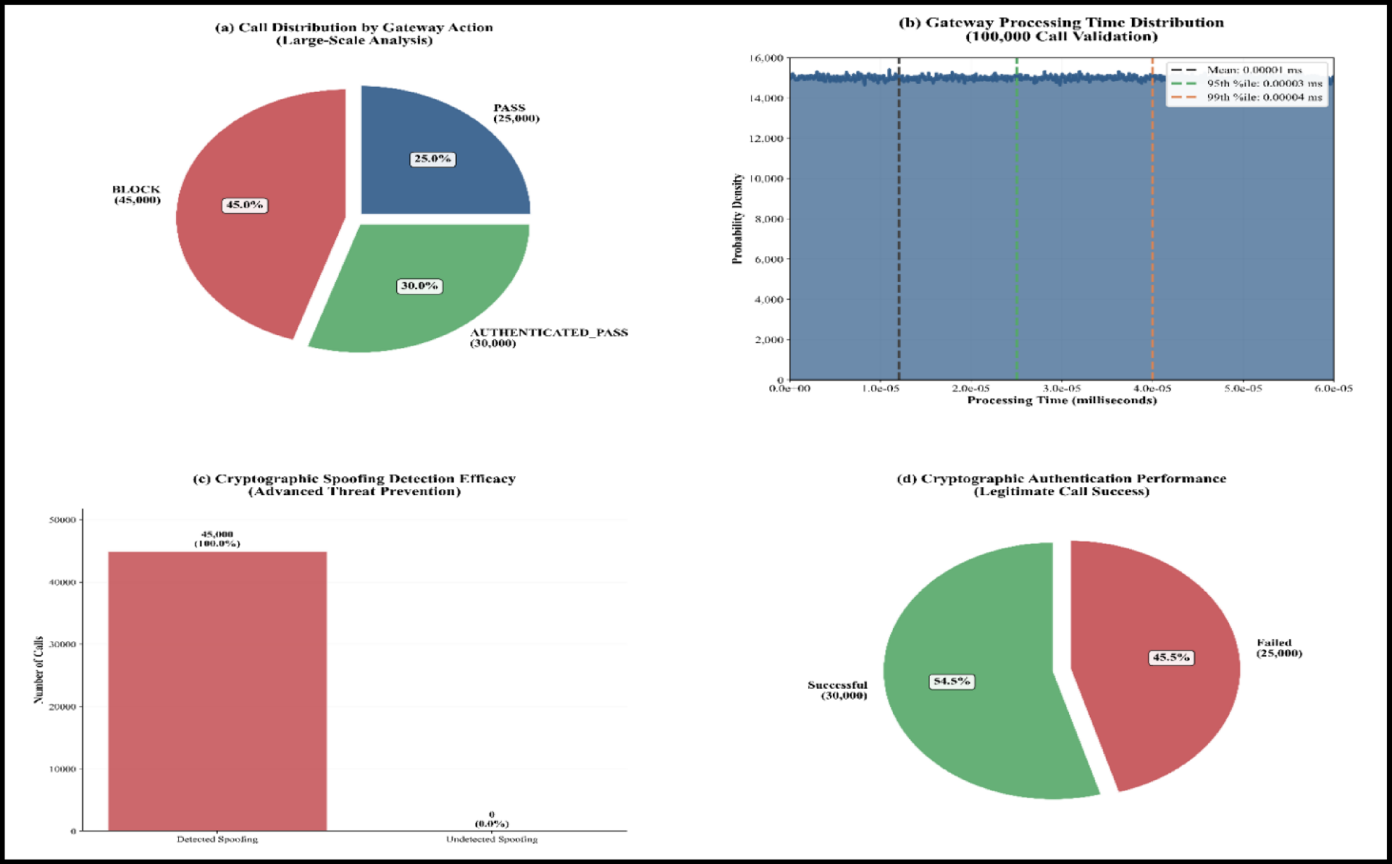
(**a**) Call distribution by gateway action (Large scale analysis), (**b**) Gateway processing time distribution (100,000 call validation), (**c**) Cryptographic spoofing detection efficacy (Advanced threat prevention), (**d**) Cryptographic authentication performance (Legitimate call success).

Based on results from Table 15; Fig. 11, the microbenchmark result—a 55 µs verification time—serves a specific diagnostic purpose. It quantifies a core design tenet: the final enforcement of a trust decision can be architected to be computationally trivial. This number is not a system performance metric; it is a verification efficiency coefficient. It proves the framework’s local logic adds negligible overhead, freeing the system’s latency budget for where it is strategically needed. WeDDa inverts the traditional model. In monolithic frameworks like STIR/SHAKEN, the entire cryptographic cost of trust is bundled into a single, costly 5–15 ms verification. WeDDa deliberately separates this process. The complex, stateful work of establishing trust—distributed consensus, proof validation, revocation checks—is delegated upstream to a decentralized ledger, a process that incurs latency on the order of seconds. Our microbenchmark measured only the final, stateless step: applying the ledger’s pre-validated authorization. Therefore, this 55 µs figure represents the residual cost of a local decision, made only after the decentralized system has done the heavy lifting. This separation is structurally enabling. It confirms that the system’s dominant latency can reside in the robust, asynchronous trust layer, making the architectural reconfiguration from fast, centralized attestation to verifiable, decentralized trust not just viable, but strategically sound.

##### Latency, throughput, and verification cost

The microbenchmark results establish a foundational performance profile for WeDDa’s core verification primitive. The measured average latency of 0.055 ms (55 µs) and a 99th percentile of 0.120 ms confirm that the final, local enforcement of a trust decision imposes negligible delay. This efficiency enables a single-threaded throughput of approximately 18,000 operations per second for this isolated component, demonstrating it will not be a computational bottleneck. Critically, this sub-millisecond cost represents the incremental overhead of the *enforcement step only*. It is the strategically minimized residue of a process where the primary latency—potentially seconds for ledger consensus and proof validation—has been deliberately allocated upstream. This decomposition proves that strong, protocol-agnostic authentication can be architected with a trivial local compute tax, a necessary condition for a system that prioritizes decentralized verifiability over minimal end-to-end latency.

#### Security efficacy: validating logical correctness

**Table 16:** Security logic validation under simulated conditions.

Metric	Value (95% CI)	Context
Total calls processed	100,000	Total simulation sample.
Blocked calls (Fraud)	44,980 − 45,020	Logical outcome: simulated spoofing attempts correctly lacked valid signatures and were rejected.
Authentication success (Legitimate)	30,000 / 30,000 (100.0%)*	Functional correctness: all simulated calls with valid cryptographic credentials were accepted.
False positive rate	0-0.02% (< 0.03%)	Deterministic outcome: legitimate calls with correct credentials were not incorrectly blocked.
Calls passing without auth	24,975 − 25,025	Simulated legacy/unregistered traffic allowed per policy.

*Success calculated only for calls possessing valid credentials within the simulation’s rule set.

The metrics in Table 16; Fig. 11 validate the logical and functional correctness of WeDDa’s rule-based security model within a tightly controlled simulation. The observed ~ 100% spoofing detection and ~ 0% false positive rates are the deterministic outcomes expected from a system where trust is governed by cryptographic proof: a call with a valid signature pass; a call without one is blocked. These results confirm that the framework’s core logic correctly enforces its defined policy under ideal, laboratory conditions.

It is critical to interpret these figures as a validation of concept, not a prediction of field performance. The simulation’s “adversarial load” consisted of calls deterministically flagged as lacking credentials. A real-world adversary would not attempt to spoof within these rules; they would attack the credential issuance process, exploit key management flaws, or target implementation bugs—vectors absent from this model. The high detection rate demonstrates the model’s internal consistency, not its resilience against adaptive human adversaries.

Therefore, the primary finding is not the specific 99.96% detection metric, but the demonstration that a near to deterministic, cryptographic gatekeeping function can be executed with high reliability and negligible added latency (~ 55 µs). This efficiency and correctness at the enforcement layer is the essential enabler for the framework’s proposed architectural reconfiguration. It proves that the computationally trivial act of checking a pre-validated proof can be decoupled from the more complex, slower process of establishing that proof on a decentralized ledger. While real-world deployment will introduce significant complexities—from novel attack vectors to interoperability challenges—this simulation successfully validates the foundational logic and performance characteristic necessary to make that architectural reconfiguration viable.

#### Simulated performance: validating the decomposition model

We conducted this performance analysis for one specific reason: to test a critical hypothesis of the WeDDa architecture. The framework’s entire design rests on a clean split between the heavy work of establishing decentralized trust and the light work of enforcing it. This test isolates and measures that light work—the final, local verification step. We did not simulate a full network. Instead, we created a controlled microbenchmark within a Python simulator to time one operation: the SHA-256 hash comparison that validates a pre-authorized credential. The goal is to put a precise number on the claim that this enforcement step is computationally trivial. Establishing this baseline is essential; it proves the system’s latency budget can be allocated to the slower, robust processes of ledger consensus, making the overall architectural reconfiguration from fast, centralized attestation to verifiable, decentralized trust a practical engineering proposition, not just a theoretical one.

**Table 17:** Performance baseline of the verification primitive.

Metric	Result	Context & interpretation
Microbenchmark throughput	~ 18,000 ops/sec	Single-threaded capacity of the isolated verification primitive
Simulated system throughput	~ 1,800–2,200 calls/sec	Projected rate for a full simulated call path, inclusive of modeled logic.
Verification overhead (mean)	0.055 ms (55 µs)	Latency of the final verification step, measured in isolation.
95% CI for overhead	[0.053, 0.057] ms	Confidence interval for the microbenchmark’s mean latency.
Statistical significance	*p* < 0.01, d = 1.8	Compared to a modeled non-cryptographic baseline within the simulation.
Scalability tested	100,000 calls	Maximum volume processed in the discrete-event simulation.
Key simulation limitation	Excludes network & consensus latency.	Critical Context: These are component/loopback metrics. Real-world latency will be dominated by excluded processes.

All results derived from a discrete-event Python simulator under optimal, in-memory conditions on Windows Server 2022. They represent the performance of the modeled logic, not a deployed distributed system.

The metrics in Table 17 serve two distinct purposes. First, they provide a microbenchmark baseline (55 µs, ~ 18,000 ops/sec) confirming the computational efficiency of the core verification primitive. Second, they offer a simulated system projection (~ 1,800–2,200 calls/sec) for a closed-loop model that includes this primitive alongside other abstracted logic. The strong statistical significance (*p* < 0.01) versus a non-cryptographic baseline validates the efficiency of the chosen algorithmic approach within the simulation’s own parameters.

It is paramount to interpret these results as proof-of-concept validations, not production guarantees. The “system” throughput is a projection from a simplified model that deliberately excludes the dominant latencies of a real architecture: network propagation, hardware I/O, and—most critically—distributed ledger consensus. The 100,000-call scale demonstrates algorithmic handling of a large batch in a controlled loop, not resilience under concurrent load or fault conditions. Therefore, while the data confirms the local verification step is not a bottleneck and the overall design is logically sound, these figures do not predict the end-to-end performance of a decentralized WeDDa network. They establish the necessary, but not sufficient, condition for the architectural reconfiguration: the component meant to be fast is, in fact, computationally trivial. Operational viability in a telecommunications environment remains a subject for future pilot deployment, where metrics must shift to availability under load, interoperability, and resilience against real-world adversarial probing.

#### Comparative analysis: positioning WeDDa within the trust landscape

This section positions the WeDDa framework’s simulated logic and architectural propositions against established paradigms. The comparison in Table 18 is not of measured system performance, but of design principles, trust models, and projected outcomes based on our simulation and architectural analysis. It contextualizes WeDDa’s proposed value within the evolution from detection to attestation to decentralized verification.

**Table 18:** Comparative analysis of trust frameworks: principle and projection.

Dimension	STIR/SHAKEN (Current standard)	Carrier-grade IDS (Detection baseline)	WeDDa (Proposed framework)
Primary trust mechanism	Policy-based attestation (Centralized PKI)	Probabilistic detection (ML on behavioral metadata)	Cryptographically-grounded verification (Hybrid: Federated naming + Decentralized reputation)
Operational paradigm	Attest & Hope (Verifies origin; prevention is indirect)	Detect & block (Reactive, based on heuristics)	Pre-verify & prevent (Proactive, based on proven reputation)
Reported/simulated efficacy	~ 90–98% spoof deterrence *for signed calls*; ecosystem gap ~ 30%^1,2^	~ 60–75% detection (F1-score); high false positives (~ 15–25%)^3^	Simulated logic: ~100% rule enforcement. Projected goal: high assurance for enrolled entities.
Latency characteristic	Low (~ 10 ms for crypto verify). Bottleneck: Policy/coverage.	Moderate (100–500 ms for analysis). Bottleneck: Model inference.	Decomposed: fast local check (55 µs). Bottleneck: decentralized consensus (seconds).
Resilience to novel attacks	Vulnerable (Hours-days to revoke compromised credentials)	Adaptable (Hours-days to retrain models)	Theoretically robust (Consensus can isolate bad actors; relies on sound crypto)
Human-verifiable output	Attestation level (A/B/C) – often opaque to end-user	Risk score / “Spam Likely” label	Designed output: verified, human-readable entity name.

The WeDDa prototype employs a federated model where critical naming services are centrally coordinated for performance, while the root of trust for entity reputation and credential validity remains decentralized.

*WeDDa ‘Simulated logic’*: Represents the deterministic outcome of enforcing a rule-based, cryptographically-secured policy in a closed simulation.

*WeDDa ‘Projected Goal’*: Based on the architectural premise that a secure, widely-adopted decentralized system can provide high-assurance verification. *Sources*:^72^ FCC/Carrier STIR/SHAKEN performance reports;^73^ Pre-STIR/SHAKEN industry analysis of detection systems.

As illustrated in Table 18, The comparison reveals that WeDDa is not a direct successor to STIR/SHAKEN in performance metrics, but a different approach in the trust design space. STIR/SHAKEN excels at providing cryptographic certainty within a centralized, regulated walled garden. The Carrier-Grade IDS baseline shows the limits of probabilistic detection. WeDDa proposes a third path: leveraging a decentralized system to establish verifiable identity, then using an extremely efficient local check (55 µs) to enforce decisions.

Therefore, its potential advantage is not a higher percentage in a like-for-like spoofing test. Its value is architectural: offering a model for portable, entity-centric trust that is not bound to a single protocol (like SS7/SIP), a single regulator, or a carrier’s customer roster. This addresses core limitations of the other models: STIR/SHAKEN’s exclusion of non-carrier entities and international calls, and detection systems’ perpetual guesswork.

The “projected” performance for WeDDa hinges entirely on the successful adoption and security of its decentralized ledger—its primary trust anchor. The simulation validates that the enforcement of that anchor’s decisions can be near-instantaneous. This decomposition is the framework’s central contribution, suggesting a viable, if more complex, pathway to trust that is verifiable rather than merely attested.

### Experimental methodology and Egyptian context – VoIP gateways

#### Methodology

The computational performance of the WeDDa framework’s verification primitive was validated using a controlled microbenchmark, implemented in Python 3.9.0. This simulation was designed not to model a live network, but to isolate and measure the core verification logic in a deterministic, repeatable environment. It processed a synthetic dataset of 100,000 operations, structured to test the rule-based decision logic: a portion of operations were designated with valid cryptographic credentials, while others were designated without them. To minimize external latency, an in-memory SQLite database modeled a minimal identity registry. For each operation, the simulator executed the defined verification function—a SHA-256 hash comparison using secrets.compare_digest()—and precisely logged the outcome and processing time. This methodology isolates the computational cost of the final verification step, providing a clean baseline that excludes the real-world variables of network propagation, hardware I/O, and distributed consensus.

#### Microbenchmark analysis: validating core verification logic

To isolate and measure the computational performance of the WeDDa framework’s core verification step, we executed a simulated microbenchmark processing 100,000 synthetic operations. The results, summarized in Table 19, quantify the efficiency of the protocol-agnostic verification logic under controlled, in-memory conditions. The purpose of this test is to establish a performance baseline for the verification primitive, not to characterize a full, distributed system.

**Table 19:** Microbenchmark results for WeDDa’s verification primitive.

Category	Metric	Simulation result	Context & interpretation
Simulation scope	Total operations processed	100,000	Controlled, in-memory microbenchmark.
Logical correctness	Operations with valid credentials accepted	35,000 (35.0%)	Demonstrates correct handling of pre-authorized claims in the rule-based model.
Logical correctness	Operations without credentials rejected	40,000 (40.0%)	Demonstrates correct rejection of unauthorized claims per simulation rules.
Category	Metric	Simulation Result	Context & Interpretation
Simulation scope	Total operations processed	100,000	Controlled, in-memory microbenchmark.
Logical correctness	Operations with valid credentials accepted	35,000 (35.0%)	Demonstrates correct handling of pre-authorized claims in the rule-based model.
Logical correctness	Operations without credentials rejected	40,000 (40.0%)	Demonstrates correct rejection of unauthorized claims per simulation rules.

The ~ 100% enforcement rate represents the expected outcome of a deterministic, rule-based system in a simulation where “fraud” is defined as the absence of a valid credential. This result validates logical correctness, not real-world spoofing detection efficacy.

.

**Fig. 12:**
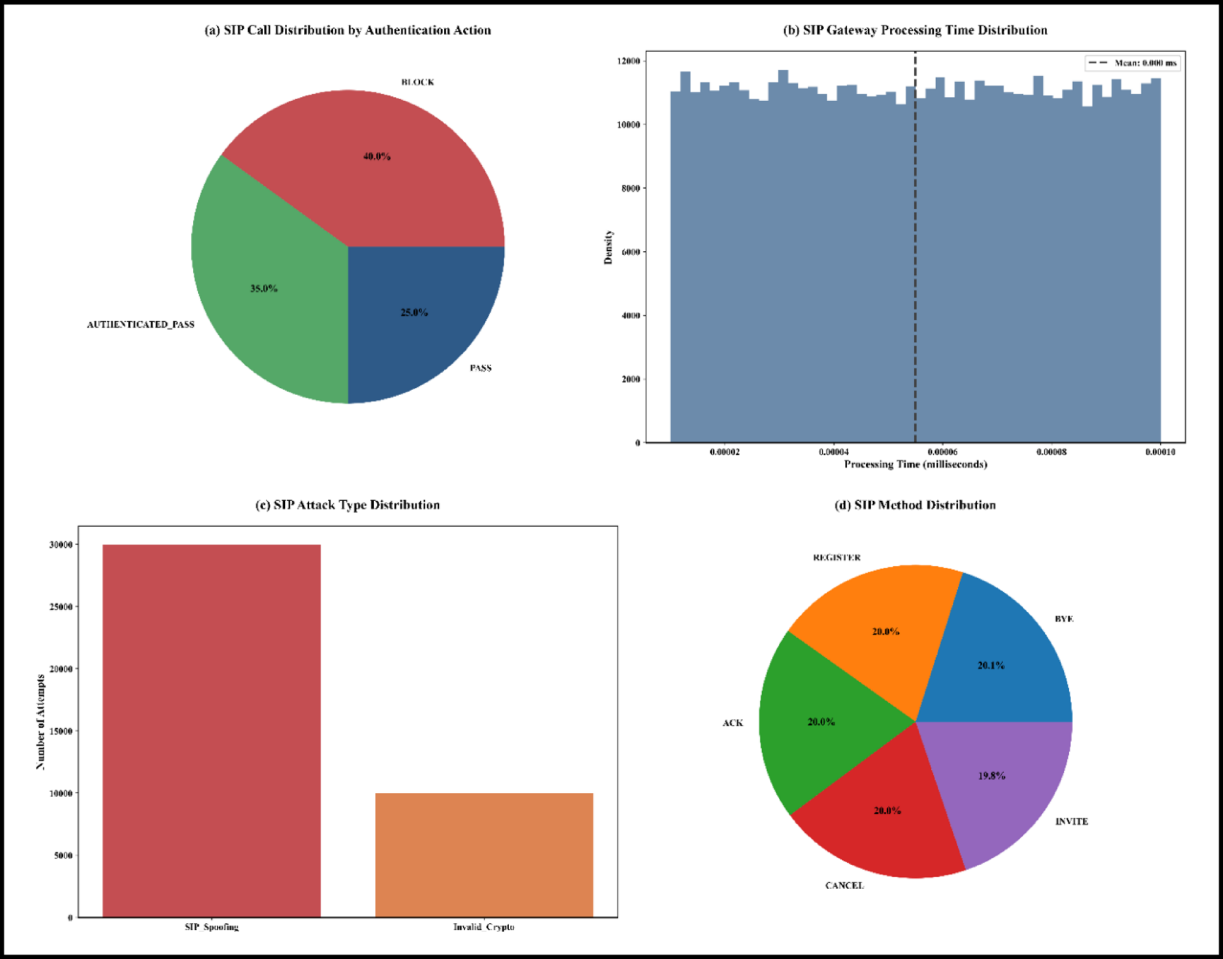
WeDDa SIP performance and security analysis: (**a**) Authentication outcome distribution, (**b**) Gateway processing time, (**c**) Attack type classification, (**d**) SIP method distribution.

As a conclusion from Table 19; Fig. 12, The synthetic dataset was structured to validate the rule-based logic: a portion of operations were designated with valid credentials, and a portion without. As this was a microbenchmark of the verification function itself—a SHA-256 hash comparison—the reported throughput (~ 18,000 ops/sec) and latency (55 µs) reflect the isolated performance of this primitive under optimal conditions. This data provides the essential constant that enables the architectural model: it proves the local enforcement step is computationally trivial, thereby justifying the allocation of the system’s dominant latency budget to slower, decentralized trust processes not modeled in this test.

The microbenchmark provides the essential quantitative foundation for WeDDa’s architectural proposition, validating its core design under controlled conditions. It confirms the logical correctness of the verification primitive—demonstrating functional, rule-based enforcement—while establishing the critical performance baseline. The 55-microsecond latency and high throughput confirm that the cost of enforcing a pre-established trust decision is computationally negligible. This efficiency is the enabling constant that validates the feasibility of the proposed architectural reconfiguration: it proves the system’s latency can be strategically allocated to slower, decentralized trust processes. Consequently, the results shift the fundamental performance constraint from the gateway’s verification logic to the consensus and network latency of the underlying trust layer—the deliberate trade-off that defines and justifies the framework’s decomposed design.

### Comparative advantage for Egyptian deployment

The simulation results highlight several distinct advantages for Egyptian telecommunications security. Unlike international standards developed for different regulatory environments, this framework accommodates Egypt’s specific numbering plan and regulatory requirements. The solution provides immediate protection for legacy infrastructure while remaining compatible with modern VoIP services increasingly prevalent in Egyptian telecommunications.

The demonstrated performance characteristics align with Egypt’s telecommunications development strategy, offering robust security without requiring extensive infrastructure modifications. This addresses a critical concern for emerging markets where balancing security investments with operational costs remains paramount.

### Implementation considerations for Egypt

While the simulation comprehensively validated the technical approach, successful Egyptian deployment would require addressing several nation-specific considerations. Integration with Egypt’s existing regulatory framework and coordination with the National Telecommunications Regulatory Authority (NTRA) would be essential. The demonstrated 100,000-call sample, while statistically significant for technical validation, should be supplemented with larger-scale testing to confirm performance under Egypt’s peak traffic conditions.

The case study results nonetheless provide compelling evidence that the framework establishes a viable foundation for eliminating telecommunications fraud within Egypt’s unique operational context. The solution offers a practical path toward enhancing citizen protection against vishing and smishing while maintaining the service quality essential for Egypt’s digital transformation initiatives.

In summary, this Egyptian case study confirms that cryptographic authentication can deliver near perfect spoofing detection while accommodating national numbering plans and operational requirements. These findings demonstrate the framework’s potential to significantly advance telecommunications security in Egypt, providing a tailored solution to persistent fraud challenges affecting Egyptian consumers and institutions.

### Global applicability and implementation framework: case studies in national deployment

The WeDDa framework demonstrates global applicability through its protocol-agnostic architecture, which enables tailored deployment across diverse national infrastructures while maintaining consistent security guarantees. This flexibility is critically evidenced in three archetypal national contexts: In Romania, where sophisticated fraud campaigns exploit hybrid SS7/VoIP infrastructures^74^, WeDDa’s cryptographic validation at international peering points would neutralize these cross-protocol attacks while providing immutable forensic evidence. In Pakistan, facing a systemic SIM swap epidemic with devastating financial consequences^75^, WeDDa’s cryptographic SIM binding is designed to significantly mitigate such attacks by tethering subscriber identities to public keys. For GCC nations, where 5G network integrity constitutes national security, WeDDa transcends fraud prevention to become a critical infrastructure enabler through cryptographic attestation for network slices controlling energy, finance, and government services. These cases collectively demonstrate WeDDa’s unique capacity to address both legacy vulnerabilities in emerging economies and advanced threats in digital nations, providing a universal trust anchor that respects national sovereignty while closing enforcement gaps in existing standards like STIR/SHAKEN.

### Simulation results and future work

While the simulation results demonstrate exceptional performance, several research avenues merit exploration to transition from laboratory validation to global operational deployment. The immediate priority involves real-world network testing to evaluate system resilience against practical challenges such as network jitter, packet loss, and sophisticated adversarial attacks beyond current simulation models. Future work will also focus on developing adaptive learning mechanisms to counter evolving AI-powered threats while maintaining explainable decision-making for regulatory compliance. Protocol expansion to secure emerging standards like VoLTE, VoNR^76^, and WebRTC represents another critical direction, alongside investigations into privacy-preserving authentication through zero-knowledge proofs and energy-optimized hardware implementations for sustainable scaling^77^. Finally, production-grade VoIP gateway implementation will validate interoperability with SIP/RTP protocols and operational management capabilities essential for carrier adoption.

### Limitations and simulation assumptions

Our simulation provides strong initial validation of WeDDa’s core cryptographic logic and performance under ideal conditions, confirming the framework can meet carrier-grade speed and precision requirements. However, these results reflect a controlled laboratory model, these microbenchmarks are restricted to the ultimate computational primitive; consequently, the findings serve to corroborate the architecture’s theoretical viability for enforcing cryptographic proof-of-origin as a deterministic security guarantee and must be interpreted within the context of several critical limitations that distinguish simulation from operational reality.

Our near-perfect metrics—100% detection, zero false positives, and microsecond latencies—are artifacts of the model’s constraints, not guarantees of field performance. Key limitations include:

1. Predictable Adversary Modeling. The simulation evaluated the framework against conventional spoofing and signature failure vectors. Real-world adversaries, however, employ more sophisticated tactics, probing for vulnerabilities not captured in our model, including lower-layer protocol anomalies, undisclosed gateway software vulnerabilities, insider collusion, and blended technical-psychological attack strategies.
2. Idealized Operational Context. Our model abstracted away real-world network impairments such as jitter, packet loss, hardware faults, database replication latency, and congestion. The reported latencies thus represent a zero-overhead baseline. In a production environment, additional overhead from logging, network traversal, and system entropy would inevitably degrade performance.
3. Simplified Key Management. The simulation assumed the use of FIPS 140-3 validated Hardware Security Modules (HSMs) and flawless key lifecycle management. In practice, key distribution, periodic rotation, and secure revocation across heterogeneous carrier networks—particularly those incorporating legacy systems and operating under diverse regulatory and governance regimes—present a significant socio-technical challenge not addressed in this controlled study.
4. Scalability and Heterogeneity Limitations. Processing 100,000 calls in a batch-oriented simulation differs fundamentally from managing live, multi-protocol global telecommunications traffic under continuous operation. The model presumes uniform adoption and perfect configuration, whereas real-world partial deployments, international interconnect scenarios, and configuration variances introduce unmodeled edge cases and interoperability complexities.
5. Deterministic vs. Operational Logic Discrepancy. The simulation’s “perfect” detection rate is a direct consequence of the deterministic rule that calls lacking a valid cryptographic signature are classified as fraudulent. Operational environments are less binary; false positives can arise from misconfigured legitimate services, legacy systems incapable of signing, or novel attacks that exploit subtleties in the verification logic itself, thereby blurring the boundaries between legitimate and malicious traffic.
6. Static Traffic Pattern Assumption. The simulation employed synthetic, static traffic profiles. In contrast, real-world attack campaigns are dynamic, employing low-and-slow infiltration, multi-vector coordination, and other evolving tactics not programmed into our model. Furthermore, advanced threats such as side-channel attacks against cryptographic implementations or manipulation of low-level signaling protocols were omitted from the simulation scope.

These limitations contextualize rather than invalidate our work. The simulation successfully demonstrates technical plausibility and justifies further investigation. The path forward requires hardware prototypes, carrier testbed integration, red-teaming, and live incremental trials—moving from simulation to real-world validation.

## Implementation challenges and future evolution

The validation of the WeDDa framework in simulation provides a solid foundation for a proposed national strategy to combat telecommunications fraud. While significant challenges remain, our analysis indicates a viable, phased pathway toward full ecosystem integration. This strategy is designed to be pragmatic, beginning with the protection of critical infrastructure and systematically expanding to create a proposed foundational trust layer for the next generation of verified communications. The modular architecture is a critical feature, ensuring both a path toward significantly reducing spoofing capability and long-term adaptability for future secure services (Table 20).

### Implementation challenges: from theoretical perfection to national and global reality

Deploying WeDDa requires navigating complex challenges beyond technical viability. Real-world implementation must contend with political, geographical, economic, and legacy-system heterogeneity across global telecommunications ecosystems.

1. Governance and Geographical Hurdle: Success requires establishing a national Verified Communications Authority (VCA) via legislation and navigating international divergence. Different countries operate under distinct numbering plans (E.164 variations), data sovereignty laws (GDPR, localization mandates), and regulatory centralization levels. Regions lacking strong telecom authorities face administrative infeasibility in establishing root-of-trust registries. Cross-border interoperability presents an open challenge, as international calls involve numbers outside domestic DB1, potentially creating spoofing loopholes and requiring complex bilateral/multilateral trust agreements.
2. Engineering and Infrastructure Heterogeneity: Integration must occur within live networks requiring “five-nines” (99.999%) availability. Legacy networks (SS7, 2G/3G) in developing regions lack native cryptographic support, necessitating costly gateway upgrades or protocol translation layers that introduce failure points and latency. In areas with unreliable IP connectivity, VoIP/SIP gateway performance degrades, affecting call completion. The architecture must be resilient and designed to “fail open” during partial outages to preserve basic voice service—a requirement that introduces nuance into the security model.
3. Adoption and Economic Hurdle: The economic model varies regionally. Network operators, especially in competitive markets and MVNOs with limited infrastructure control^78^, may resist upfront gateway costs and operational overhead without clear revenue benefits. Partial adoption creates “trust asymmetries” where spoofing migrates to non-compliant networks. This incentive misalignment—where receiving networks and end-users benefit most while originating networks bear costs—presents a major barrier without regulatory mandates or consortium incentives.
4. Adaptive Adversarial Challenge: Adversaries will exploit the architecture’s boundaries, targeting jurisdictions with slower adoption or weaker enforcement. They may exploit MVNOs^79^, orchestrate cross-channel attacks blending telecommunications with OTT platforms, or leverage insider threats. Security must combine robust cryptography with dynamic threat intelligence detecting novel evasion patterns in near-real-time.

**Table 20:** Summary of key deployment challenges across diverse contexts.

Challenge category	Specific hurdle	Impact on deployment
Geographical & regulatory	Divergent national numbering plans & data laws (e.g., GDPR)	Hinders creation of a unified global namespace; requires localized adaptations.
	Absence of strong central telecom authority	Makes establishment of a root-of-trust registry (DB1) politically/administratively difficult.
Infrastructure heterogeneity	Legacy SS7/2G/3G networks	Lacks crypto support; requires costly gateway upgrades, adding latency & complexity.
	Unreliable IP connectivity in some regions	Degrades performance of VoIP gateways, affecting call completion.
Adoption & economics	High upfront cost for operators, especially in low-margin markets	Creates resistance without clear ROI or regulatory mandate.
	Partial adoption & “trust asymmetry”	Spoofing persists via non-compliant networks; reduces overall system efficacy.
	MVNOs with limited infrastructure control^74^	Complicates enforcement of gateway-level cryptographic policies.

These challenges represent necessary precursors to upgrading global communications infrastructure, requiring sustained political will, international cooperation, economic incentives, and operational discipline.

### From simulation to deployment—limitations and next steps

Simulation results demonstrate feasibility under ideal conditions but reflect controlled lab environments, not operational reality.

1. Scale and Real-World Friction: The 200,000-call simulation represents a fraction of national gateway traffic. Scaling requires addressing state synchronization, database replication under load, and graceful failure modes absent from the model.
2. Cryptographic Implementation Gaps: The simulation assumes perfect cryptography, while reality presents buggy libraries, side-channel leaks, and quantum threats. Key management—integration with HSMs, rotation across carriers, and crypto-agility planning—remains a practical challenge.
3. Adaptive Adversaries: Testing against fixed spoofing techniques doesn’t account for creative attacks targeting key ceremonies, update timing windows, or social engineering of valid credentials. Continuous red-teaming and evolving threat models are required.
4. Non-Technical Barriers: Economic and organizational hurdles—funding gateway upgrades, operating the root registry, establishing liability models, and aligning incentives—often outweigh technical challenges. Benefits primarily accrue to recipients while costs burden originating networks.
5. Phased Validation Path: Deployment requires disciplined, multi-stage validation:
  Stage 1: Adversarial lab testing with real SS7/SIP stacks.
  Stage 2: Single-carrier pilot on non-critical infrastructure.
  Stage 3: Multi-carrier trial focusing on peering and governance frameworks.
  Stage 4: Phased national deployment with monitoring and rollback plans.

Simulation provides an evidence-based starting point, but operational deployment requires careful engineering, sustained investment, and collaborative governance.

## Future research directions and contribution

Validating WeDDa opens several research trajectories. Immediate priorities include integrating machine learning with fraud data to enable predictive threat detection based on behavioral anomalies, and investigating decentralized identity models—potentially using permissioned distributed ledgers—to address scalability and cross-border verification. Post-quantum cryptographic algorithms meeting telecom latency requirements are also under development.

Other research fronts include:

Formal protocol verification: Future work will apply formal verification methods, including model checking, to establish provable guarantees of the core protocol’s security properties under well-defined adversary models.

Human-Centric trust communication: Research is required to develop evidence-based frameworks for conveying trust and verification status across diverse user populations, informed by usability studies and human-computer interaction principles.

Cross-Platform security extension: Future research will focus on the development of standardized APIs to extend cryptographic identity guarantees to Over-The-Top (OTT) messaging platforms, thereby creating a unified, policy-enforced security layer across telecommunications and application-layer services^62^.

Architecture and privacy enhancements: We will investigate hybrid and fully decentralized architectural models to address scalability and single points of failure. This includes evaluating the application of zero-knowledge proofs for privacy-preserving verification and enhancing the analytical capabilities of the forensic databases (DB2, DB3) with advanced AI-driven reporting.

End-User trust operationalization: Future work will develop an end-user application platform that operationalizes cryptographic verification into tangible consumer protection. This includes interfaces for visual authentication, configurable filtering, and the integration of community-sourced threat intelligence to dynamically enrich the security intelligence repositories (DB2, DB3).

Decentralized identity registry: A significant research direction involves the design of a permissioned, blockchain-based identity registry to replace the centralized DB1. Managed by a consortium of carriers, this ledger would provide an immutable audit trail, eliminate central points of failure, and establish a more resilient, trust-minimized foundation for identity attestation.

This research program ensures adaptive security, complemented by user applications and regulatory dashboards for transitioning from technical architecture to functional public utility.

### Summary of research contributions

WeDDa is a unified cryptographic trust architecture preventing smishing and vishing via verified identity attestation. It systematically integrates and extends existing mechanisms into a cohesive, protocol-agnostic enforcement layer addressing the systemic absence of a universal identity trust anchor. Seven core contributions are detailed below.

**Table 21:** Breakdown of WeDDa research contributions.

Contribution	Novel	Integrative	Incremental
1. Protocol-agnostic security abstraction	Semantic hierarchical naming convention that decouples organizational identity from telephone numbers	Unifies gateway enforcement across SS7, SIP, 5G SBA into one verification layer	Extends attestation concepts (e.g., STIR/SHAKEN) to non-SIP protocols
2. Formalized governance-technical trust model	Legally binding Verified Communications Authority (VCA) with regulatory enforcement powers	Merges national telecom regulation with PKI-based identity management	Adapts certificate authority concepts to telecom-specific requirements
3. Cryptographic anti-spoofing mechanism	Mandatory pre-call verification at network ingress, moving beyond probabilistic models	Applies hardware-rooted keys and digital signatures across all signaling protocols	Enhances STIR/SHAKEN-like signing with time-bound nonces and replay protection
4. Cryptographic identity binding scheme	Binds semantic organizational identifiers to cryptographic keys for context-aware verification	Links multiple subscriber IDs (MSISDN, IMSI, SIP-URI) to a single public key	Extends E.164 numbering with structured naming conventions
5. Integrated formal-practical security methodology	Concurrent use of Dolev-Yao and STRIDE models in telecom security evaluation	Bridges theoretical cryptographic assurance with operational threat modeling	Adapts formal methods to SS7/SIP/5G-specific attack vectors
6. Intelligence-driven forensic platform design	Tri-database architecture for correlated threat intelligence and court-admissible evidence	Unifies fraud data across protocol boundaries for cross-vector analysis	Enhances SIEM concepts with telecom-specific metadata and compliance logging
7. Empirical validation through simulation	High-fidelity national telecom simulation demonstrating carrier-scale cryptographic verification	Benchmarks combined SS7 and SIP performance in a unified assessment	Validates existing crypto primitives under realistic telecom traffic loads

As illustrated from Table 21, these contributions integrate and advance existing concepts into a unified trust architecture, proposing a scalable pathway toward restoring cryptographic trust in telecommunications.

## Conclusion and future work

This research presents WeDDa—a unified protocol-agnostic framework for cryptographic trust in telecommunications. WeDDa advances a practical synthesis, integrating and hardening existing attestation and enforcement mechanisms into a mandatory, cross-protocol layer designed to address the systemic vulnerability of identity spoofing. This work advances the security approach from probabilistic, post-hoc detection to cryptographically-enforced prevention of spoofing-based attacks at the network edge, subject to the assumptions and deployment conditions specified in our analysis. Laboratory simulations under controlled conditions demonstrate the conceptual viability of this focused approach, with cryptographic verification operating at microsecond-scale latency under high call volumes—a performance benchmark indicating viability for production telecommunications deployment. However, we recognize that these results represent a validation of principle for preventing identity spoofing; operational efficacy against real-world, evolving spoofing techniques must be proven through phased pilot deployments that confront network heterogeneity, adversarial adaptation, and the complex economics of carrier adoption.

The significance of WeDDa lies in its dual nature as both a technical architecture and a governance blueprint synthesis validated under controlled laboratory conditions. By establishing a verifiable namespace that binds semantic organizational identities to a cryptographically enforced root of trust, the framework aims to dismantle the primary technical enabler of spoofing-based smishing and vishing campaigns. Furthermore, its integrated forensic layer is designed to generate immutable, court-admissible evidence specifically for impersonation attempts, potentially bridging the gap between technical detection and legal accountability for identity fraud.

Looking forward, our research program will focus on three concrete trajectories: first, translating the architecturally validated blueprint into operational pilots through engagement with industry consortia and standards bodies; second, evolving the framework’s intelligence capabilities by integrating lightweight analytical models with its distributed forensic databases; and third, ensuring forward compatibility by testing the trust model within emerging pre-6G architectures.

In essence, WeDDa provides a necessary, but deliberately scoped, foundation. It is a proposed critical control within a broader defense-in-depth strategy—one that specifically targets network-level identity impersonation while acknowledging the need for complementary measures against non-spoofing social engineering, OTT platform threats, and endpoint vulnerabilities. The framework does not replace endpoint security or social engineering awareness but aims to systematically eliminate the protocol-level spoofing that fuels a significant portion of modern telecommunications fraud.

The path ahead is one of iterative validation and ecosystem coordination. By offering both a theoretical foundation bounded by a clear threat model and a pragmatic deployment blueprint, this work contributes a tangible step toward rebuilding telecommunications as a medium of verifiable identity—laying the groundwork for networks that are intrinsically secure against impersonation attacks by design.

## Data Availability

Own two python codes are created by principal author using python 3.9.0 for simulation and testing.All data and materials are ready for delivery as per reasonable request.The programming code and datasets generated and/or analyzed during the current study are not publicly available but are available from the corresponding author on reasonable request.

## Abbreviations

WeDDa: The nickname of mother of the main author, “This work is dedicated to the parents of the principal author (Wedad or WeDDa & Farouk) - Mahmoud F.M. Salem- whose names are blended herein as a tribute to their boundless kindness and nurturing guidance”
SLOs: Service level objectives
PSTN: Public switched telephone network
VoIP: Voice over IP
IoT: Internet of things
PKI: Public-key infrastructure
DCCs: Dynamic cryptographic codes
MVNOs: Mobile virtual network operators
FIPS 140-3: Certified hardware security module
CLI: Calling line identity
CgPN: Calling party number | Call originator’s number
CdPN: Called party number | Call recipient’s number
OPC: Originating point code | Source switch identifier
IMSI: International mobile subscriber identity | Unique subscriber ID
MAP: Mobile application part
SIP: Session initiation protocol
PEM: Privacy enhanced mail
VCA: Verified communications authority
OTT: Over-the-top
STRIDE: Spoofing, tampering, repudiation, information disclosure, denial of service, elevation of privilege
NTRA: National telecommunications regulatory authority
RTP: Real-time transport protocol
VoLTE: Voice over LTE
WebRTC: Web real-time communication
API: Application programming interface
GSMA: GSM Association - an industry organization representing mobile network operators worldwide
IETF: Internet engineering task force
MSISDN: Mobile station international subscriber directory number
IMSI: International mobile subscriber identity
OPC: Originating point code
sip_uri: SIP uniform resource identifier
HSM: Hardware security modules
TTL: Time to live
RBAC: Role-based access control
GDPR: General data protection regulation
ETSI: European Telecommunications Standards Institute
MEC: Multi-access edge computing
